# Evolution and dispersal of mitochondrial DNA haplogroup U5 in Northern Europe: insights from an unsupervised learning approach to phylogeography

**DOI:** 10.1186/s12864-022-08572-y

**Published:** 2022-05-07

**Authors:** Dana Kristjansson, Jon Bohlin, Truc Trung Nguyen, Astanand Jugessur, Theodore G. Schurr

**Affiliations:** 1 Center for Fertility and Health, Norwegian Institute of Public Health, Oslo, Norway; 2 Department of Global Public Health and Primary Care, Faculty of Medicine, University of Bergen, Bergen, Norway; 3 Department of Method Development and Analytics, Norwegian Institute of Public Health, Oslo, Norway; 4 IT Systems Bergen, Norwegian Institute of Public Health, Bergen, Norway; 5 Department of Anthropology, University of Pennsylvania, Philadelphia, PA USA

**Keywords:** Scandinavia, Migration, Phylogeny, Clade, Haplotype

## Abstract

**Background:**

We combined an unsupervised learning methodology for analyzing mitogenome sequences with maximum likelihood (ML) phylogenetics to make detailed inferences about the evolution and diversification of mitochondrial DNA (mtDNA) haplogroup U5, which appears at high frequencies in northern Europe.

**Methods:**

Haplogroup U5 mitogenome sequences were gathered from GenBank. The hierarchal Bayesian Analysis of Population Structure (hierBAPS) method was used to generate groups of sequences that were then projected onto a rooted maximum likelihood (ML) phylogenetic tree to visualize the pattern of clustering. The haplogroup statuses of the individual sequences were assessed using Haplogrep2.

**Results:**

A total of 23 hierBAPS groups were identified, all of which corresponded to subclades defined in Phylotree, v.17. The hierBAPS groups projected onto the ML phylogeny accurately clustered all haplotypes belonging to a specific haplogroup in accordance with Haplogrep2. By incorporating the geographic source of each sequence and subclade age estimates into this framework, inferences about the diversification of U5 mtDNAs were made. Haplogroup U5 has been present in northern Europe since the Mesolithic, and spread in both eastern and western directions, undergoing significant diversification within Scandinavia. A review of historical and archeological evidence attests to some of the population interactions contributing to this pattern.

**Conclusions:**

The hierBAPS algorithm accurately grouped mitogenome sequences into subclades in a phylogenetically robust manner. This analysis provided new insights into the phylogeographic structure of haplogroup U5 diversity in northern Europe, revealing a detailed perspective on the diversity of subclades in this region and their distribution in Scandinavian populations.

**Supplementary Information:**

The online version contains supplementary material available at 10.1186/s12864-022-08572-y.

## Significance statement

We wanted to explore the genetic structure of haplogroup U5 in northern Europe by employing an unsupervised learning approach for phylogenetic clustering. We accurately identified groups of mitochondrial DNA (mtDNA) sequences that were mapped onto a phylogenetic tree in order to make historical inferences about human population history. Our results support previous hypotheses that haplogroup U5 mtDNAs expanded throughout Northern, Southern and Central Europe with more recent expansions into Western Europe and Africa. The results further allow us to explain how U5 mtDNAs are now found with high frequency in Northern Europe, as well as delineate the origins of the specific U5 subhaplogroups found in that part of Europe. We have found that the integration of hierBAPS clustering with a maximum likelihood phylogenetic analysis delineates clusters of similar sequences at sufficient resolution to allow broader inferences about lineage diversification and population migration history to be made from them. To our knowledge, this combined approach has not yet been applied to studies focused on human mtDNA diversity

## Introduction

Over the past three decades, mitochondrial DNA (mtDNA) variation has been used to trace human ancestry in population genetic studies. The mtDNA is particularly informative for evolutionary studies because it represents a non-recombining part of the human genome, is maternally inherited, and evolves at a clock-like rate [1]. For this reason, many tens of thousands of mitogenomes from different human populations have been sequenced in an effort to reconstruct the phylogeographic history of our species.

Since its first build was published in 2008, Phylotree has become one of the most comprehensive libraries of known global human mtDNA variation [2]. It provides a systematic haplogroup nomenclature based on signature polymorphisms observed in the published sequences entered in the database. Until recently, Phylotree has been continually updated with newly available mitogenome sequences, and currently incorporates data from 24,275 mitogenomes [2].

Despite it being a valuable resource, the nomenclature used in Phylotree to assign a haplogroup status to individual sequences remains tedious and prone to errors. This is especially the case when a haplogroup must be assigned to several sequences from a human population in which several branches of an ancestral haplogroup may have slightly varying mutations. Although algorithmic software that incorporates Phylotree nomenclature can aid in haplogroup identification [3–7], constructing a phylogenetic tree that is consistent with Phylotree haplogroup labeling still remains an iterative and slow process.

A maximum likelihood (ML) phylogeny based on single nucleotide polymorphism (SNP) calling can be referentially rooted at an ancestral sequence, and also take into account character transformations using different evolutionary models that can be validated using bootstrapping methods or bootstrap approximations [8–10]. While ML is often employed to understand the evolutionary relationship of non-human species, its use in human mtDNA analyses has been limited due to the tediousness of assigning each mitogenome sequence to a Phylotree haplogroup. In addition, the similarity of the sequences in large human populations typically studied in these analyses can often result in unintelligible, dense, and unorganized trees. As a consequence, the genetic relationships of groups of similar sequences become difficult to disentangle and categorize for broader, evolutionary inferences. Furthermore, since Phylotree was last updated back in February 2016, several haplogroups have been recently defined but not integrated into the current nomenclatural system [11–15]. Thus, a method that could quickly categorize new sequences at high resolution would be extremely useful for phylogenetic studies.

One such method of making these classifications is the hierarchical Bayesian Analysis of Population Structure (hierBAPS) algorithm. This algorithm identifies clusters of sequences based on the corresponding allele frequencies within that cluster [16]. It is especially useful for quickly grouping sequences from several individuals who have different haplotypes but share a common ancestral lineage. The grouping of large clusters of ancestrally derived sequences further allows broader inferences to be made about their evolution, and can lead to a more refined visual organization that may not be evident based on detailed haplogroup labeling alone.

The hierBAPS clustering has usually been conducted in studies of haploid DNA from microorganisms [17, 18]. In particular, it has been utilized for several years in combination with ML phylogenetics for studies of bacterial populations [16, 19, 20]. However, this combined methodology has yet to be applied to an evolutionary analysis of human mtDNAs.

### Haplogroup U5 as a case study

Haplogroup U5 is one of the most ancient mtDNA lineages to have existed in Central Europe prior to its dispersal into Northern Europe [21, 22]. This haplogroup is thought to have evolved in the western steppe region [23] and then entered Europe around 30 to 55 kya [1, 24]. It appears to have expanded into Europe before the end of the Last Glacial Maximum (LGM) over 20 thousand years ago (kya) [1, 25–27], i.e., before the thick ice sheets covering most of northern continental Europe were in the final stages of dissipating away from the interior.

Today, the frequency of U5 varies between 5–12% in most European countries [28, 29]. Its frequency varies particularly widely within Northern Europe. Haplogroup U5 mtDNAs are present in northern Saami populations at over 50% [30–32], while their corresponding frequencies in the southern areas of the Scandinavian countries (Norway, Sweden, and Denmark) lie between 6–15% [31, 33, 34]. These differing frequencies raise interesting questions about the phylogenetic structure of this major lineage and the timing of the dispersal of its subbranches within the European continent.

On this note, while both Saami and Finns speak Finno-Uralic languages, the two populations do not share a close genetic relationship based on nuclear DNA marker loci [35]. This pattern is also true to some extent based on mtDNA data. Apart from Scandinavia, U5b mtDNAs with the “Saami motif” (defined by the T16144C, T16189C, and C16270T control-region variants; Tambets et al. 2004) have been observed at significant frequencies in populations from the northwestern Pskov Oblast and the Republic of Karelia in Russia [31, 36]. This distribution points the emergence of U5b mtDNA in ancestral Saami (Uralic) groups, and their dispersal into surrounding Indo-European populations through admixture.

Based on this evidence, it is generally agreed that the Saami are genetically distinct from other European populations [32, 37, 38], although the source of U5 mtDNAs among these European populations is not entirely clear. Therefore, a broader analysis of the phylogeographic features of haplogroup U5 is necessary to fill this knowledge gap. The aim of this study is thus to combine hierBAPS analysis of haplogroup U5 mitogenome sequences with maximum likelihood (ML) phylogenetics to make inferences about the evolution and dispersal of this major maternal lineage in Northern Europe.

## Materials and methods

### Mitogenome sequences

Data for haplogroup U5 mitogenome sequences were retrieved from the European Nucleotide Archive and GenBank (*n* = 873) (accessed on 31 May 2021) and the search was limited to “whole mtDNA” and “haplogroup U5”. For the purposes of this study, we separated Nordic populations into Saami, Scandinavia (Norway, Denmark, and Sweden), and Finland categories. Finland was separated from Scandinavia in this analysis due to its geographic isolation from the Scandinavian Peninsula and its linguistic distinctiveness. Specific information about the ethnicity or original location of the individuals represented by these sequences was available for 855 (97.8%) of the total dataset. The accession numbers of the samples are provided in the data availability statement.

### Phylogenetic analysis

#### Maximum-likelihood phylogeny

We constructed a ML phylogeny from the 873 U5 mitogenome sequences with the software IQ-tree 1.6.12 [9]. The phylogeny was constructed under the best fitting nucleotide substitution model inferred by jModelTest [39, 40], which was TIM3 + F + R3 based on the Bayesian Information Criterion (BIC). Branch support was achieved by the approximate likelihood ratio test (aLRT) [41] based on resampling the estimated log-likelihood method with a simple but effective collection scheme of candidate trees [39]. This was accomplished by applying the UFBoot algorithm [10] for 10,000 replicates. UFBoot overcomes the computational burden required by the standard nonparametric bootstrap, and can be interpreted as providing an unbiased bootstrap support with 95% support which corresponds to a 95% probability that a clade is true [42].

#### Partitioning mtDNA sequences using hierBAPS

To identify clusters of closely linked sequences within the 873 U5 mitogenome sequences, we employed the hierBAPS algorithm [43]. This algorithm groups DNA sequences into clusters in a hierarchical manner, and can be used to project the grouped sequences onto an independently derived phylogenetic tree [19]. The hierBAPS algorithm assumes that each individual sequence is drawn from one of several distinct genetic subpopulations, with each cluster having its own set of allele frequencies.

To apply hierBAPS to mtDNA sequences, we utilized an R software implementation of algorithm, RhierBAPS, that is available on the Comprehensive R Archive Network [19]. Briefly, the hierBAPS algorithm attempts to maximize the posterior probability of an allocation of a sequence over other possible allocations, assigning each individual sequence to specific clusters. After the number of clusters (*K*) is specified, the algorithm partitions the sequences of the dataset into as many groupings as possible (up to *K*_max_ clusters). The initial number of *K* clusters can be chosen based on the number of subpopulations expected, and can be increased on each re-run of the algorithm. The algorithm is typically re-run until the number of clusters stops increasing.

The clusters were refined into levels of low to high resolution of cluster specificity. We conducted three different cluster-level combinations: *Level 1*: 4 groups, *Level 2*: 11 groups, and *Level 3*: 24 groups. To distinguish Phylotree labels from hierBAPS groups for the demonstrative purposes of this study, alphabetical letters or roman numerals were used to represent hierBAPS labels. It is important to note here that the hierBAPS group labels provided by the algorithm, denoted by roman numerals, are generated in arbitrary order.

We also explored hierBAPS clustering using only the coding regions of the mitogenome sequences. This step was carried out by extracting the coding regions of the sequences using the Harvesttools package [44]. We conducted four cluster-level combinations on these data: *Level 1*: 3 groups, *Level 2*: 6 groups, *Level 3*: 12 groups, and *Level 4*: 18 groups. The highest resolution results for both the coding region only and the whole mitogenome sequences were then compared.

#### Haplogroup identification

We used Haplogrep, version 2.1.21 [5] to assign a haplogroup to each mitogenome sequence based on its mutational signature, independent of the hierBAPS grouping. Haplogrep computes these classifications on pre-calculated phylogenetic weights that correspond to the occurrence of a polymorphism per position in Phylotree Build 17 [2], which, in turn, reflects the mutational stability of a variant. Mutations were identified relative to the Reconstructed Sapiens Reference Sequence (RSRS) [24], which allows for the naming and mapping of human mtDNA haplogroups from an ancestral root.

To be clear about the outcome of this analysis, we have utilized the following definitions when discussing the details of the U5 phylogeny. First, a *haplogroup* is a group of similar haplotypes that share a combination of ancestral polymorphisms commonly inherited together, such as U5. Similarly, a *subhaplogroup* is a branch of a haplogroup containing a subset of the sequences defined by the parent haplogroup but defined by its own set of mutation, such as U5b or even more specifically U5b1b1b. By contrast, a *subclade* is a cluster of related haplotypes associated with a hierBAPS grouping. On a more general level, a *lineage* is a maternal line of descent often referred to in population studies, and a *branch* is a part of the phylogenetic tree that extends from a root or major trunk.

#### Haplogroup age estimates

A temporal framework for the divergence of haplogroup U5 branches was assessed with TempEst v.1.5.1 [45]. Age estimates with 95% confidence intervals were calculated using the Least Squares Dating IQ-tree plugin [46]. To calibrate the ages, we used a root age based on the reported 177 ± 11 kya age estimation for the RSRS sequence reported by Behar and colleagues [24], as well as radiocarbon dating for ancient samples bearing U5 mtDNAs [22, 47, 48].

### Comparative data analysis

Due to the fact that the GenBank sequences were collected for specific research purposes, had a low sample size per region, and did not encompass all geographic locations, it was not possible to make conclusions about haplogroup prevalence based solely on these data. Thus, the GenBank sequences were only utilized in this study for the purposes of making conclusions about the groupings and evolutionary relationships between sequences from an ancestral inference point.

To understand the geographical prevalence of U5 based on more representative data, we conducted a search of studies reporting the frequency of U5 mtDNAs within various populations. The frequency from each specific region was then tabulated. For more specific information about the major subhaplogroups within U5, we obtained data from 6488 individuals from the public database on the U5 mtDNA Project available from FamilyTreeDNA [49]. The overall frequencies of U5 mtDNAs were plotted on a geographic heat map using the statistical programming language R, version 3.6.3 (The R Foundation), and its graphical package ggplot2.3 [50].

## Results

### Bayesian Analysis of hierBAPS Groups

The least detailed hierBAPS analysis (*Level 1*) identified four major clusters within haplogroup U5. These included A: U5a1; B: U5a2; C: U5b1 + U5b3; and D: U5b2. The most detailed hierBAPS analysis (*Level 3*) identified 24 groups. The 24-group analysis listed the RSRS separately as group VIII, while the other 23 groups corresponded to the specific subclades listed in Table 1. Excluding the RSRS sequence, each of the 23 hierBAPS groups shared a set of polymorphisms that enabled the hierBAPS algorithm to generate specific clusters for them (Table 2). About 32.5% (*n* = 28) of the group-defining polymorphisms occurred in the non-coding control region of the mitogenome sequence.

**Table 1:** HierBAPS groups and their representative subclade(s) based on the human mtDNA U5 haplogroup

*hierBAPS Groups*	*Broad haplogroup (4-digits)*	*Major Subclade(s) or Haplogroups*	*Specific Haplogroups Included*	*N*	*%*
*I*	U5a2	U5a2, U5a2b, U5a2c, U5a2d	U5a2, U5a2 + 16294 T, U5a2b, U5a2b1a, U5a2b1b, U5a2b1c, U5a2b1d, U5a2b2, U5a2b2a, U5a2b3, U5a2b3a, U5a2b3a1, U5a2b4, U5a2b4a, U5a2c, U5a2c1, U5a2c3a, U5a2c4, U5a2d, U5a2d1, U5a2d1a	90	10.3
*II*	U5a2	U5a2e	U5a2e	11	1.3
*III*	U5a1	U5a1, U5a1g, U5a1i	U5a1, U5a1b, U5a1b + 16362C, U5a1b1, U5a1b1a, U5a1b1b, U5a1b1b1, U5a1b1c, U5a1b1c1, U5a1b1c2, U5a1b1d + 16093C, U5a1b1d1, U5a1b1e, U5a1b1g, U5a1b1h, U5a1b2, U5a1b3, U5a1b3a, U5a1b3a1, U5a1b4, U5a1d, U5a1d1, U5a1e, U5a1f1a, U5a1f2, U5a1g, U5a1g1, U5a1i, U5a1i1 U5a1j	128	14.6
*IV*	U5b1 + U5b3	U5b1, U5b1a, U5b1d, U5b1f, U5b1i, U5b3	U5b1, U5b1a, U5b1d1a, U5b1d1b, U5b1d1c, U5b1d2, U5b1f, U5b1f1, U5b1f1a, U5b1i, U5b3, U5b3a1a, U5b3a2, U5b3b1, U5b3b2, U5b3e, U5b3h	44	5
*V*	U5b1 + U5b3	U5b1 + 16189C!, U5b1b, U5b1c	U5b1 + 16189C!, U5b1b, U5b1b2, U5b1b2a, U5b1b2b, U5b1c, U5b1c1a, U5b1c1a1, U5b1c2, U5b1c2a, U5b1c2b	72	8.2
*VI*	U5a1	U5a1a2	U5a1a2, U5a1a2a, U5a1a2a1, U5a1a2a1a, U5a1a2b1	26	3
*VII*	U5a1	U5a1h	U5a1h	7	0.8
	*RSRS*	*RSRS*	*RSRS*	1	*0.1*
*IX*	U5a1	U5a1d2	U5a1d2a, U5a1d2a1, U5a1d2b	18	2.1
*X*	U5a1	U5a1c	U5a1c	28	3.2
*XI*	U5a1	U5a1a1	U5a1a1, U5a1a1a, U5a1a1b, U5a1a1c, U5a1a1d, U5a1a1h, U5a1a1i	89	10.2
*XII*	U5b2	U5b2a	U5b2a, U5b2a1b, U5b2a3, U5b2a3a, U5b2a4, U5b2a4a, U5b2a5, U5b2a5a, U5b2a6	25	2.9
*XIII*	U5b2	U5b2, U5b2c	U5b2, U5b2c1, U5b2c2, U5b2c2b	11	1.3
*XIV*	U5b2	U5b2a2	U5b2a2, U5b2a2a1, U5b2a2b, U5b2a2b1, U5a2a2c	29	3.3
*XV*	U5b2	U5b2b	U5b2b, U5b2b2, U5b2b3a1a, U5b2b4, U5b2b4a, U5b2b5	19	2.2
*XVI*	U5b2	U5b2b1	U5b2b1a, U5b2b1a1, U5b2a1a2, U5b2b1b	10	1.1
*XVII*	U5b1 + U5b3	U5b1b1a	U5b1b1a, U5b1b1a1, U5b1b1a1a, U5b1b1a1a1, U5b1b1a1b, U5b1b1a2, U5b1b1a3	83	9.3
*XVIII*	U5b1 + U5b3	U5b1b1	U5b1b1, U5b1b1 + 152C!, U5b1b1b, U5b1b1d, U5b1b1e, U5b1b1f, U5b1b1g1, U5b1b1g1a	39	4.5
*XIX*	U5b2	U5b2a1a + 16311 T!	U5b2a1a + 16311 T!, U5b2a1a1, U5b2a1a1a, U5b2a1a1d	32	3.7
*XX*	U5b2	U5b2a1a2	U5b2a1a2	5	0.6
*XXI*	U5a2	U5a2a	U5a2a, U5a2a1, U5a2a1 + 152C!, U5a2a1a, U5a2a1b, U5a2a1b1, U5a2a1c, U5a2a1e	70	8
*XXII*	U5a2	U5a2a2a	U5a2a2a	8	0.9
*XXIII*	U5b1 + U5b3	U5b1e1	U5b1e1, U5b1e1a	25	2.9
*XXIV*	U5b1 + U5b3	U5b1e1 (+ T8337C)	U5b1e1 (+ T8337C)	6	0.7

**Table 2:** Shared mtDNA polymorphisms per hierBAPS group^a^

		hierBAPS Group													
		I	II	III	IV	V	VI	VII		IX	X	XI	XII	XIII	XIV
		U5a2, U5a2b, U5a2c, U5a2d	U5a2e	U5a1,U5a1g, U5a1i	U5b1, U5b1a, U5b1d, U5b1f, U5b1i, U5b3	U5b1 + T16189C! U5b1b, U5b1c	U5a1a2	U5a1h	*RSRS (Ancestral)*	U5a1d	U5a1c	U5a1a1	U5b2a	U5b2, U5b2c	U5b2a2
Number of shared mitochondrial polymorphisms within each U5 hierBAP group (n)		19	21	16	18	19	29	45	*1*	20	28	21	27	33	35
**Nucleotide Position**	Location														
146	HVS-II		T					T	T		T		T	T	T
150	HVS-II			T				T	C				T	T	T
151	HVS-II		T						C						
152	HVS-II		C!	T			T	T	T						T
195	HVS-II		T				T	T	T						T
247	HVS-II	G	G	G			G	G	G		G	G	G	G	G
523	HVS-III							A	A					A	
524	HVS-III							C	C					C	
769	12S_rRNA	G	G	G	G	G	G	G	G	G	G	G		G	G
1303	12S_rRNA							A	G						
1700	16S_rRNA						C		T			C			
1721	16S_rRNA								C				T	T	T
2757	16S_rRNA								A						
3027	16S_rRNA								T	C					
3107	preserves historical genome annotation numbering						d	d	-						d
3192	16S_rRNA							T	C						
3197	16S_rRNA	C	C		C	C	C	C	T	C	C	C	C	C	C
3212	16S_rRNA								C						T
3552	ND1 (Ala—3rd position in codon)								T	C					
3591	ND1 (Leu—3rd position in codon)							A	G						
3768	ND1 (Leu—3rd position in codon)		G						A						
4592	ND2 (Ser—3rd position in codon)							C	T						
4732	ND2 (Asn—2nd position in codon)								A				G		G
5452	ND2 (Thr—2nd position in codon)								C						
5495	ND2 (Phe—3nd position in codon)								T			C			
5656	position between tRNA-Ala and tRNA-Asn					G			A						
7146	CO1 (Ala—1st position in codon)	A	A	A	A	A	A	A	A		A	A		A	A
7256	CO1 (Asn—3rd position in codon)	C	C	C	C	C	C	C	C	C	C	C	C	C	
7521	tRNA-Asp	G	G		G	G	G	G	G	G	G	G	G	G	G
7768	CO2 (Met—3rd position in codon)				G	G			A				G	G	G
7853	CO2 (Val—1st position in codon)								G						
8337	tRNA-Lys								T						
8701	ATP6 (Ala—1st position codon)	A	A	A		A	A	A	A	A	A	A	A	A	A
8705	ATP6 (Met—2nd position codon)								T						
9477	CO3 (Val—1st position codon)	A	A	A	A	A	A	A	G	A	A		A	A	A
9540	CO3 (Leu—1st position codon)	T	T	T	T	T	T	T	T	T	T	T	T	T	T
10,283	ND3 (Leu—3rd position codon)								A						
10,398	ND3 (Ala—1st position codon)	A	A	A	A	A	A	A	A	A	A		A	A	A
10,810	ND4 (Leu—3rd position codon)	T	T	T	T	T		T	T	T	T	T	T	T	T
10,873	ND4 (Pro—3rd position codon)	T	T		T	T	T	T	T	T	T	T	T	T	T
10,915	ND4 (Cys—3rd position codon)		T	T	T	T	T	T	C	T	T	T	T	T	T
10,927	ND4 (Phe—3rd position codon)								T						
11,296	ND4 (Leu—3rd position codon)							T	C						
11,653	ND4 (Val—3rd position codon)								A						
11,914	ND4 (Thr—3rd position codon)	G	G		G		G	G	G	G	G		G	G	G
11,938	ND4 (Leu—3rd position codon)							T	C						
12,308	tRNA-Leu		G	G		G	G	G	A	G	G	G	G	G	G
12,346	ND5 (His—1st position codon)						T		C						
12,372	ND5 (Leu—3rd position codon)	A	A	A		A	A	A	G	A	A	A	A	A	A
12,406	ND5 (Val—1st position codon)								G						
12,616	ND5 (Leu—1st position codon)								T						
12,618	ND5 (Leu—3rd position codon)							A	G						
12,634	ND5 (Ile—1st position codon)								A						
12,705	ND5 (Ile—3rd position codon)								C						
13,105	ND5 (Val—1st position codon)	A		A	A	A		A	A	A	A	A	A	A	A
13,145	ND5 (Ser—2nd position codon)								G						
13,276	ND5 (Val—2nd position codon)								A						
13,617	ND5 (Ile—3rd position codon)		C	C		C	C	C	T	C	C	C	C	C	C
13,630	ND5 (Thr—1st position codon)								A						
13,637	ND5 (Gln—2nd position codon)								A				G	C	G
14,182	ND6 (Val—1st position codon)					C			T				C		C
14,518	ND6 (Gly—1st position codon)								A						
14,793	CYB (His—2nd position codon)	G	G				G	G	A	G	G				
15,218	CYB (Thr—1st position codon)						G	G	A	G	G				
15,497	CYB (Gly—1st position codon)								G						
15,511	CYB (Asn—3rd position codon)								T						
15,924	tRNA-Thr								A						
16,114	HVS-I								C						
16,129	HVS-I				G			G	G			G	G	G	G
16,187	HVS-I	C	C		C		C	C	C					C	
16,189	HVS-I						T		T			C!	T!!		
16,192	HVS-I		T					T	C			T		T	
16,223	HVS-I	C	C		C		C	C	T		C			C	C
16,230	HVS-I	A			A		A	A	G		A	A	A	A	A
16,239	HVS-I							T	C						
16,256	HVS-I		T					T	C		T				
16,270	HVS-I								C					T	T
16,278	HVS-I		C				C	C	C		C		C	C	C
16,294	HVS-I								C						
16,311	HVS-I		C!					T	T		T			T	T
16,320	HVS-I								C		T				
16,362	HVS-I								T						
16,398	HVS-I								G						A
16,399	HVS-I						G	G	A		G				
16,465	HVS-I								C						
16,519	HVS-I							T	T						

	hierBAPS Group									
	XV	XVI	XVII	XVIII	XIX	XX	XXI	XXII	XXIII	XXIV
	U5b2b	U5b2b1	U5b1b1a	U5b1b1	U5b2a1a + C16311T!	U5b2a1a2	U5a2a	U5a2a2a	U5b1e1	U5b1e1 (+ T8337C)
Number of shared mitochondrial polymorphisms within each U5 hierBAP group (n)	33	36	20	32	32	41	18	40	33	38
**Nucleotide Position**										
146	T	T		T	T	T		T	T	T
150	T	T		T	T	T			T	T
151										
152						T		T		
195	T	T			T	T		T		
247	G	G		G		G		G	G	G
523		A		A		A		A		
524		C		C		C		C	d	d
769	G	G	G	G	G	G	G	G	G	G
1303										
1700										
1721	T	T			T	T				
2757									G	G
3027										
3107						d		d		d
3192										
3197	C	C		C	C	C	C	C	C	C
3212										
3552										
3591										
3768										
4592										
4732					G	G				
5452						T				
5495										
5656			G	G					G	G
7146	A	A	A	A	A	A	A	A	A	A
7256	C	C	C	C	C	C	C	C	C	C
7521		G	G	G	G	G	G	G	G	G
7768	G	G	G	G	G	G			G	G
7853								A		
8337										C
8701	A	A	A	A	A	A	A	A	A	A
8705						C				
9477	A		A	A	A	A	A		A	A
9540	T	T	T	T	T	T		T	T	T
10,283									G	G
10,398	A	A	A	A	A	A	A	A	A	A
10,810	T	T	T	T	T	T	T	T	T	T
10,873	T	T	T	T	T	T	T	T	T	T
10,915	T	T	T	T	T	T	T	T	T	T
10,927				C						
11,296										
11,653	G	G								
11,914	G	G	G	G	G	G		G	G	G
11,938										
12,308	G	G	G	G	G	G	G	G	G	G
12,346										
12,372	A	A	A	A	A	A	A	A	A	A
12,406								A		
12,616									C	C
12,618			A	A						
12,634	G									
12,705							C			
13,105	A	A	A	A	A	A	A	A	A	A
13,145								A		
13,276							A			
13,617	C	C	C	C	C	C	C	C	C	C
13,630	G	G								
13,637	G		C		G	G				
14,182	C	C		C	C	C			C	C
14,518								G		
14,793							G	G		
15,218										
15,497		A								
15,511					C	C				
15,924						G				
16,114								A		
16,129	G	G				G		G	G	G
16,187	C	C		C	C	C		C	C	C
16,189		T			T!!	T		T		
16,192		C!				C!			C!	C!
16,223	C	C		C	C	C		C	C	C
16,230	A	A		A	A	A		A	A	A
16,239										
16,256								T		
16,270		T						T	T	T
16,278	C	C		C	C	C		C	C	C
16,294								T		
16,311	T			T	C!			T		T
16,320										
16,362										
16,398										
16,399										
16,465								T		T
16,519										T

^a^ The Ancestral state is represented by the RSRS sequence. Blank cells indicate that the nucleotide position was not a factor in determining the hierBAPS group. All BAPS groups also contain the following mutations: 825 T,1018G,2758G,2885 T,3594C,4104A,4312C,8468C,8655C,10664C,10688G,11467G,12705C,13276A,13506C,13650C. Mutations are reckoned in forward evolutionary time direction in reference to the RSRS sequence. In case of a transversion, the derived allele is shown in lowercase instead of uppercase. Exclamation mark signifies back mutation to the ancestral sequence RSRS. (!) for single mutation and (!!) for double back mutation. Yellow-colored boxes indicate mutations that are diagnostic for particular haplogroup or subclade as per Phylotree. *ATP* ATP synthase, *CO* Cytochrome c oxidase, *CYB* Cytochrome b

All hierBAPS groups and the specific set of polymorphisms shared among them were mutually exclusive, i.e., no haplogroups were defined by a set of polymorphisms that was common to two different hierBAPS clusters. Additionally, the hierBAPS algorithm was able to accurately cluster all sequences belonging to a specific subhaplogroup even though each member of a hierBAPS group did not contain all diagnostic polymorphisms for a haplogroup defined by Phylotree. For example, not all sequences clustering in subclade III, represented by subhaplogroup U5a1, contained the polymorphisms 14793G and 16256 T, which are diagnostic for this subhaplogroup according to Phylotree, build 17. However, all subclade III sequences contained a sufficient number of common polymorphisms unique to them such that they could be partitioned to this branch within the U5 phylogeny.

The hierBAPS analysis also revealed considerable substructure within subhaplogroup U5b. Subhaplogroup U5b3, which is present in less than 1% in most human populations [51], was placed in subclade IV along with several other U5b1 sequences. Despite having other differences between them, the haplotypes within subclade IV shared two specific control region mutations, 16233C and 16230A, which caused them to cluster together both in the ML phylogenetic tree and in subclade IV.

By contrast, the sequences assigned to subclade V appeared in two places in the phylogenetic tree. One was situated between subclades IV and XXIII, and the other between subclades XXIV and XVIII. This subclade is also part of subhaplogroup U5b1, although all its constituent subhaplogroups (e.g., U5b1b, U5b1c) arose after the T16189C! mutational event. Subhaplogroup U5b1b1 was placed in subclade XVIII, while its daughter branches in U5b1b1a were clustered into subclade XVIII.

In addition, the hierBAPS algorithm grouped subhaplogroup U5b1e1 + T8337C (subclade XXIV) with its parent haplogroup U5b1e1 (subclade XXIII). This distinction was not previously noted in Phylotree (Build 17). Both subclades XXIII and XXIV contained a set of polymorphisms diagnostic for subhaplogroup U5b1e, with subclade XXIV sequences also having the T8337C polymorphism in the mtDNA tRNA^Lys^ gene.

### ML phylogenetic tree projection

The hierBAPS group results were projected onto an ML tree from lowest to highest number of clusters (Figure S1). The *Level 3*:24 group analysis provided the most detailed hierBAPS groups, and specific subclades could be identified in accordance with the nomenclature in Phylotree.

**Fig. 1:**
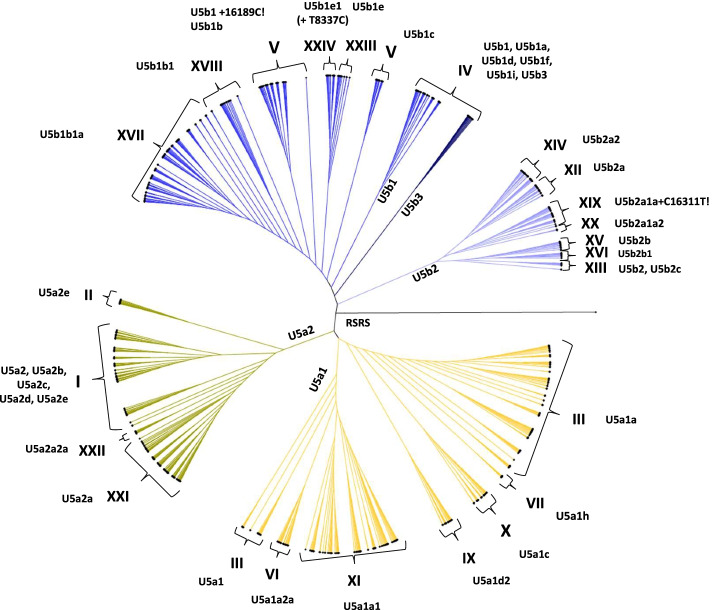
A phylogenetic tree with 23 hierBAPS groups of haplogroup U5 mitogenome sequences. Roman numerals denote the hierBAPS subclades. The hierBAPS subclades were superimposed on a phylogenetic tree, generated using maximum likelihood analysis, to help visualize the phylogenetic relationships of each sequence. The yellow coloring represents the U5a subhaplogroup while the blue coloring represents the U5b subhaplogroup. RSRS is the Reconstructed Sapiens Reference Sequence

The subclades represented by each of the 23 hierBAPS groups were mapped onto a ML phylogeny to determine how well they cohered with the phylogenetic branches produced with this method (Fig. 1). These branches could be subdivided into four main clusters guided by the *Level 1*:4 group analysis: A (U5a1), B (U5a2), C (U5b1 + U5b3), and D (U5b2). Within these main clusters, subclades with nested groups were III (U5a1) and V (U5b1 + T16189C! + T16192C!, U5b1b, U5b1c). Subclade III also had several nested subclades, including VI (U5a1a2a), XI (U5a1a1), IX (U5a1d2), X (U5a1c), and VII (U5a1h), while subclade V consisted of the nested subclades XXIII (U5b1e) and XXIV (U5b1e1 + T8337C).

The ML phylogeny generated from only the coding region of the mitogenome sequences had a similar conformation to that based on whole mitogenome sequences (Figure S2). However, the hierBAPS algorithm was able to identify more specific hierBAPS groups (*n* = 23) for the whole mitogenome sequences compared to the ML phylogeny based on coding-region sequences (*n* = 18). The coding-region hierBAPS groups and their corresponding whole mitogenomes equivalents are shown in Table S1. This table indicates that the hierBAPS groupings are less specific without the non-coding region of the mitogenome sequence.

### Geographic distribution of haplogroup U5 and its subclades

**Fig. 2:**
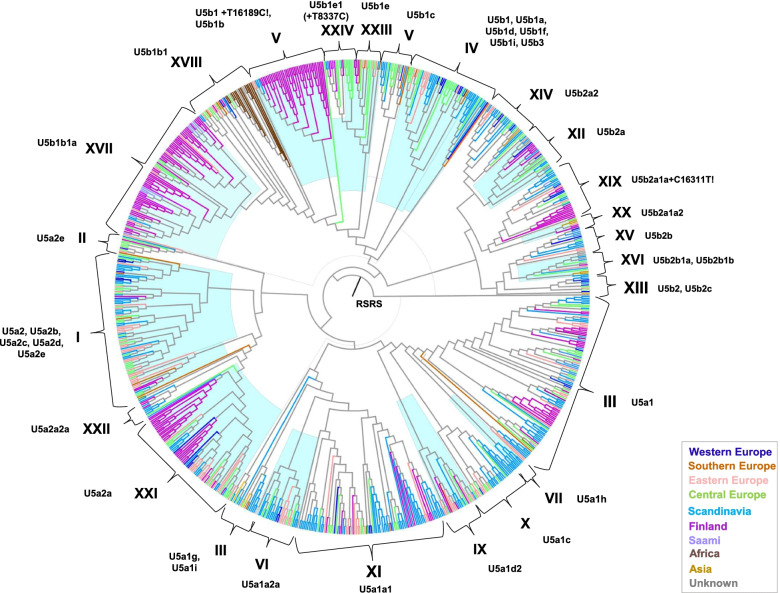
A phylogenetic tree of haplogroup U5 mitogenome sequences. hierBAPS groups are separated by a light blue watercolor. Roman numerals denote the hierBAPS subclades and their representative Phylotree based subhaplogroups. The geographic regions are defined as: Africa (Burkina Faso, Berber, Fulbe, Fulani), Western Europe (Ireland, Germany, United Kingdom), Southern Europe (France, Italy, Spain, Sardinia), Scandinavia (Denmark, Norway, Sweden), Finland, Saami (includes Saami from Scandinavia and Finland), Central Europe (Czech Republic, Hungary (Roma), Poland, Serbia, Slovenia, Slovakia), Eastern Europe (Baltic, Belarus, Caucasus, Russia), Asia (India, Iran). Unknown origins are colorized in grey

To better understand how the U5 phylogeny related to the geographical sources of the mitogenome sequences comprising it, we marked the geographic region from which each mtDNA originated using different colors (Fig. 2). The geographical distribution of the sequences is tabulated in Table S2. For the purposes of this study’s focus on northern Europe, the regions are defined by geographic location as follows: Africa (Burkina Faso, Berber, Fulbe, and Fulani ethnic groups), Western Europe (Ireland, Germany, United Kingdom), Southern Europe (France, Italy, Spain, Sardinia), Scandinavia (Denmark, Norway, Sweden), Finland, Saami (includes Saami from Scandinavia and Finland), Central Europe (Czech Republic, Hungary (Roma), Poland, Serbia, Slovenia, Slovakia), Eastern Europe (Baltic, Belarus, Caucasus, Russia), Asia (India, Iran).

Although this phylogenetic tree cannot be interpreted as an exhaustive representation of every known U5 sequence, it nevertheless provided important insights into the way that the hierBAPS groups, each representing U5 subhaplogroups, are regionally related. It also demonstrated that the hierBAPS algorithm, along with ML phylogenetic visualization, can be utilized as a starting point for understanding the divergence of mtDNA haplogroups in evolutionary and geographical terms.

### Haplogroup U5b

The phylogenetic groupings produced with the hierBAPS algorithm demonstrated that some sequences specifically clustered by geographic region (Table S3). For example, subclade V contained Central European and Scandinavian branches, including subhaplogroup U5b1c (Age: 12.1 kya; 95% CI:7.7–19.7), and subhaplogroups U5b1 + 16189C! and U5b1b (Ages: 17.6; 95% CI: 10.4–25.9 and 15.4; 95% CI: 19.5–23.2, respectively) (Table S4). Subhaplogroup U5b1e1 (age: 6.4; 95% CI: 4.2–10.1) mtDNAs were also mainly present in Central and Eastern European populations [25]. By contrast, subhaplogroup U5b1e1 sequences were nested between two branches containing Finnish and Scandinavian/Central European mtDNAs, respectively, implying that they were related to both of them.

Subhaplogroup U5b1 branched off between subclade XVIII, which includes 33% of sequences from Africa (subhaplogroup U5b1b1) (age: 12.5 kya; 95% CI: 8.8–18.2) and subclade XVII, comprised of mostly Saami and Finnish mtDNAs (subhaplogroup U5b1b1a) (age: 4.1 kya; 95% CI: 2.7–6.2) sequences. The shared ancestry of U5b1b1 mtDNAs in both the Saami and African populations confirmed findings from an earlier study suggesting that the divergence of these subhaplogroups occurred in southwestern Europe in the Franco-Cantabrian refuge during the Last Glacial Maximum [52]. Subclade XVIII sequences later spread to other African ethnic groups, including the Fulbe, Mande, and other nomadic or pastoral peoples which were part of the former Ghana Empire of Western Africa [53].

**Fig. 3:**
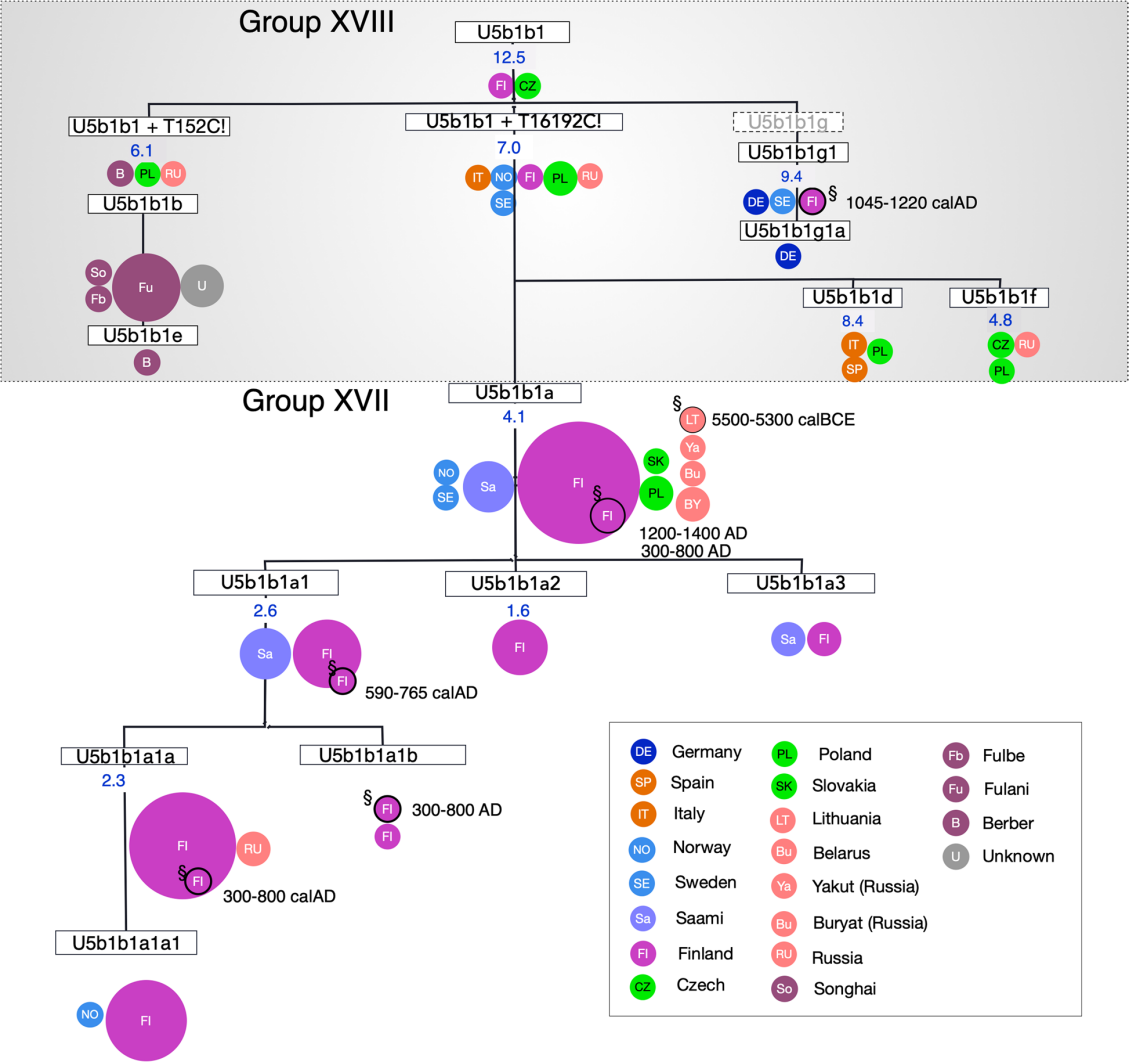
A phylogeny of the hierBAPS subclade XVII (subhaplogroup U5b1b1a) with the haplogroup U5 phylogenetic tree). The phylogeny shows detailed branching for sequences by country or ethnic origin. Time estimates kya are shown for mtDNA subhaplogroups (see Table S6). Blank ages indicate that the confidence intervals (CIs) extend to the present day. For clusters older than 200 years old (encircled in black border), the estimated rate is based on calibrated age in years before present (BP) provided by the literature. The size of the circle is proportional to the number of sequences of the same subhaplogroup, with the smallest size corresponding to one sequence. Colors indicate geographic region as in Fig. 2: Western Europe (dark blue), Southern Europe (orange), Scandinavia (light blue), Finland (magenta), Saami (lilac), Central Europe (fluorescent green), Eastern Europe (salmon), Asia (mustard)

A detailed overview of subclade XVII (subhaplogroup U5b1b1a), including the phylogenetic results and the countries in which they occur, is shown in Fig. 3, with age estimate confidence intervals being shown in Table S5. U5b1b1a is found in Finns, Saami, Poles, Belarussians, and Yakuts of eastern Russia, although the vast majority of these mtDNAs appear in the Saami and Finns. While a number of U5b1ba and U5b1b1a1 haplotypes in the Saami and Finns are similar, the Saami have U5b1b1a3 mtDNAs with the A16335G mutation that Finnish populations lack, suggesting they arose in this ethnic group.

We also found other U5 branches within Northern Europe has an estimated age older than subclade XVII (subhaplogroup U5b1b1a) (age: 4.1 kya; 95% CI: 2.7–6.2). For example, subclade XII (subhaplogroup U5b2a) (age: 22.8 kya; 95% CI: 16.3–32.1) mtDNAs were shared by Finns and Scandinavians, while subclade XXI (subhaplogroup U5a2a) (age 17.5 kya; 95% CI: 11.7–25.6) were shared by Finns and Saami. This pattern suggests the presence of subhaplogroups other than U5b1b1 among Saami populations, which may have arrived with populations from Finland or the Scandinavian peninsula.

### Haplogroup U5a

Subclade VII (subhaplogroup U5a1h) (age: 1.4 kya; 95% CI: present day-3.7), which includes 45 common polymorphisms, exhibited haplotypes with the diagnostic G1303A, C3192T, T3591A, T4592C, C11296T, C11938T, G12618A, and C16239T motif as well as other polymorphisms which are present in other U5a1 mtDNAs. Subclade U5a1h was present in six samples from Denmark and one from Yorkshire, England, indicating a probable maternal lineage of Viking Age Danish settlers in northwestern England [54].

**Fig. 4:**
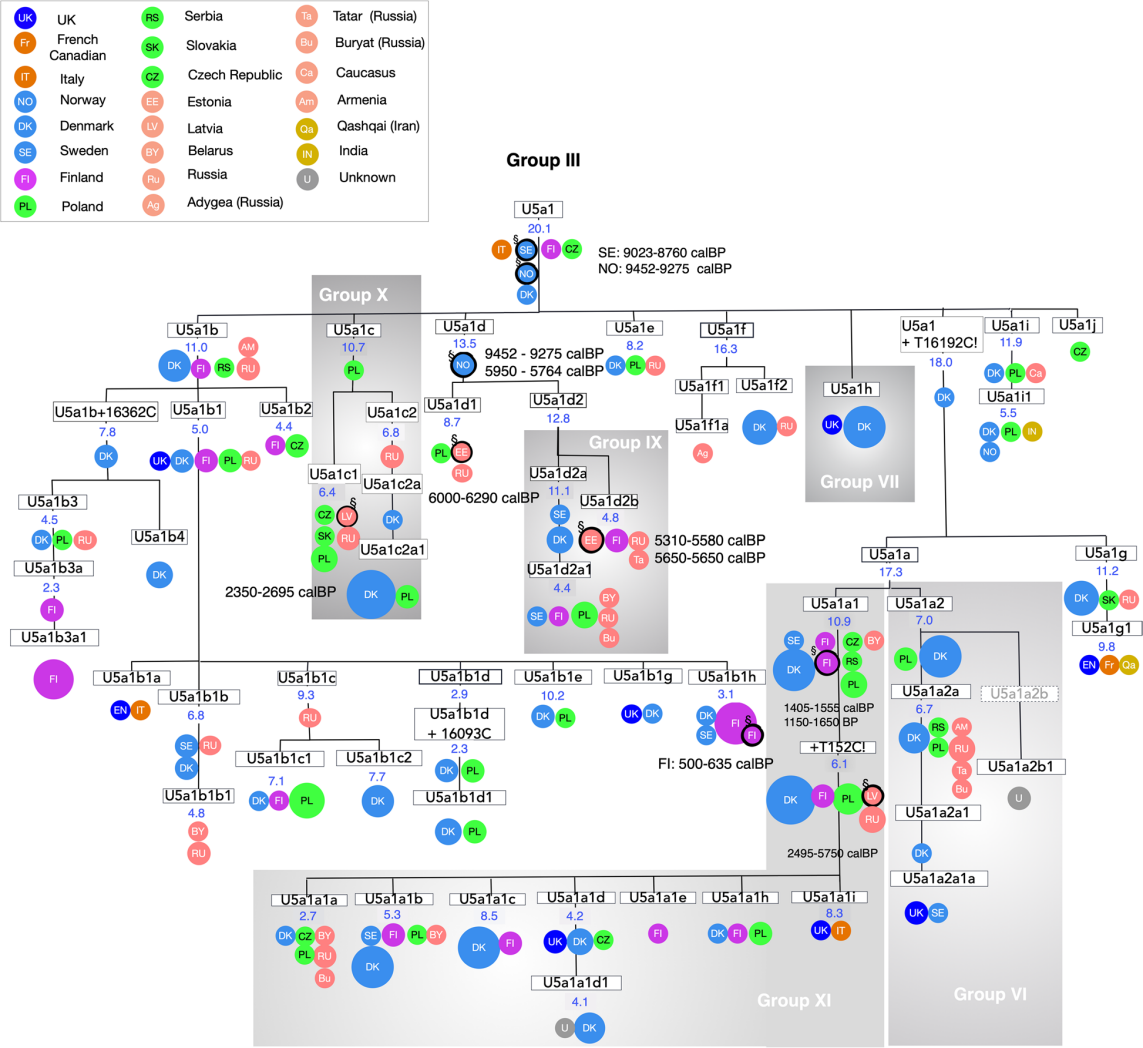
Phylogeny of the hiers BAPS subclade III (subhaplogroup U5a/U5a1) in the U5 phylogenetic tree). The phylogeny shows detailed branching for each sequence by country or ethnic origin. Time estimates are provided in kya (Table S7). Blank ages indicate confidence intervals (CIs) that extend to the present day. For clusters older than 200 years old (encircled in black border), the estimated rate provided is based on calibrated age in years before present (calBP) provided by the literature. The size of the circle is proportional to the number of sequences of the same subhaplogroup, with the smallest size corresponding to one sequence. Colors indicate geographic region as in Fig. 2: Western Europe (dark blue), Southern Europe (orange), Scandinavia (light blue), Finland (magenta), Saami (lilac), Central Europe (fluorescent green), Eastern Europe (salmon), Asia (mustard)

As expected, hierBAPS groups that occurred earlier in the phylogenetic tree were less geographically specific than later-occurring hierBAPS groups. Subclade III (Age: 20.1; 95% CI: 15.3–28.3), was the most geographically diversified hierBAPS group, and included U5a1 mtDNAs from Southern Europe, Scandinavia, Finland, Central Europe, and Eastern Europe (Fig. 4; Table S6). Subclade X (subhaplogroup U5a1c) (Age: 10.7 kya (95% CI: 5.7–18.1), contained mostly sequences from Eastern and Central Europe with some coming from Denmark. The sequences of U5a1b (Age: 11.0 kya (95% CI: 8.0–16.3) contained several geographic regions, with its distal haplotypes being mostly Scandinavian, Finnish and Eastern European in origin, A similar trans-European clustering was observed for haplogroup U5a1a1 (subclade XI) (Age: 10.9 kya (95% CI: 7.8–16.0)).

Interestingly, subhaplogroups U5a1g (Age: 11.2 kya (95% CI: 6.5–18.6) and U5a1i (Age: 11.9 kya (95% CI: 6.2–19.8), which are found in Iran (Qashqai), India, the Caucasus, and Russia, point to the dispersal of some U5a lineages into eastern regions, as well. These subhaplogroups lacked the extended daughter lineages observed in other subhaplogroups of U5a1. This finding suggested that these lineages did not diversify as successfully as did U5a1b and U5a1a, or else the current sampling of global populations is sufficiently incomplete so as not to reveal any derivative branches. In either case, there is also a lack of daughter haplogroups for U5a1i and U5a1g in Phylotree [2].

### Comparative data analysis

**Fig. 5:**
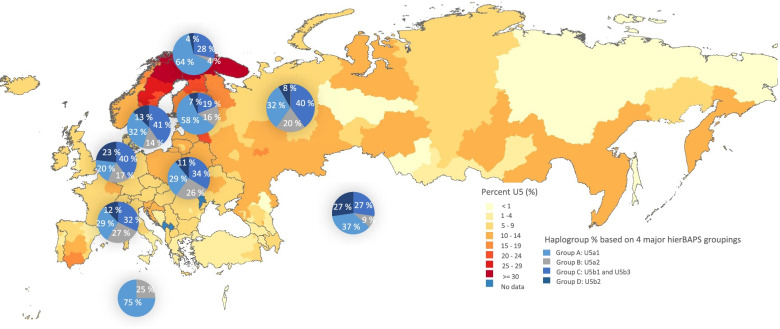
The frequency of haplogroup U5 mtDNAs in global populations based on the literature (see Table S3). The proportions of each subhaplogroup are listed, based on the four major hierBAPS groups from the FamilyTreeDNA’s U5 project. The sample sizes for each data set were as follows: Western Europe (*n* = 537), Scandinavia (*n* = 397), Sami (*n* = 78), Finland (*n* = 344), Southern Europe (*n* = 124), Central Europe (*n* = 166), and Eastern Europe (*n* = 157). Countries within Asia (*n* = 11) and Africa (*n* = 4) were combined due to their small sample sizes, with the los frequency of U5 mtDNAs being supported by the literature (Table S3)

To obtain further information about the dispersal of U5 mtDNAs, we aggregated sequence data for this haplogroup from published sources (Table S7 and Supplemental Material 1) and projected them onto a Eurasian map (Fig. 5). Overall, U5 mtDNAs were most prevalent among Saami populations of Norway, Sweden, Finland, and the Kola Peninsula (between 40–64.8%). They were next most frequent in Uralic speakers, mostly Finns (23.1% in higher latitudes of Finland to 15.6% in the southernmost part of the country) [27, 31, 35, 55], and then Estonians, Karelians (16.0%) [31], Mordovians (15.9%) [31], and Russians from the Pskov Oblast (19.2%) [36], the latter region having long barrow burials pointing to early Finnic tribe settlements in the ninth-tenth century [56]. In addition, north-dwelling Norwegians (19.0%) [57–60] and Swedes (16.6%) [31, 55] had a moderate frequency of U5 mtDNAs. As a whole, Finns had a higher proportion of U5 mtDNAs than Scandinavians [37].

We found almost exclusively U5b1 sequences (9/10) in the Saami, and this finding is consistent with previous studies showing that the majority of Saami U5 sequences belonged to this subhaplogroup (about 40–65%, depending on the country) [30, 32, 61]. Even so, we observed a single Saami sequences in subclade XXI (U5a2a), which appears to have separate evolutionary origin from those from the younger subclade XVII (U5b1b1a). It is therefore possible that U5 mtDNAs in the Saami have two sources, the first being Southern Europe via the Franco-Cantabrian refuge (U5b1), and the other from Finland and/or Central Europe (U5a2) [subclade XXI]). With regard to the U5a2 sequence, it was detected in a Saami from Finland, and may have entered Northern Europe during 8^th^ to ninth century migrations from Estonia [62].

In populations from Western, Southern and Central Europe, none of the four major subhaplogroups (U5a1, U5a2, U5b1, U5b2) represented more than 50% of the U5 mtDNAs found in those regions. This distribution implies that a greater diversity of U5 subhaplogroups is present in these areas. Since U5a has been most prevalent in Mesolithic Eurasia at approximately 65% [28, 48] and appears to be widespread, it is less clear as to whether this subhaplogroup had a west-to-east or east-to-west dispersal. Since we found evidence of its earliest haplogroups across Europe, it is more likely that dispersal happened in both directions.

Subhaplogroup U5b1b diverged and spread in different directions from Europe. According to our survey of GenBank sequences and the results of Achilli and colleagues [52], nearly all African U5 sequences belong to subhaplogroup U5b1b. Its dispersal across North Eurasia and into North Africa suggests that U5b1 had the broadest dispersal of the U5 subhaplogroups.

## Discussion

When applied to a dataset of 873 human U5 mitogenome sequences, a combination of hierBAPS clustering with ML analysis accurately reconstructed phylogenetic branches that were consistent with the haplogroup U5 phylogeny presented in Phylotree. The findings support the view that the spread of U5 mtDNAs in Northern Europe was skewed from west-to-east through U5b, although some subhaplogroups of U5a found in Northern Europe appear to have been dispersed in both west-to-east and east-to-west directions.

Compared to using Haplogrep2 alone, the hierBAPS groups provide a less tedious, yet accurate method for clustering several haplogroups to investigate population history questions requiring multiple levels of analytical refinement of mtDNA haplogroups. For population genomics, in which several individual sequences are considered simultaneously, this method of mitogenome sequence characterization provides an additional layer for identifying nested genetic population structures separated by allelic patterns. Combining hierBAPS with an ML tree also allows an understanding of similar groups from an evolutionary inference point. To our knowledge, this is the first study to incorporate a hierBAPS analysis with ML phylogenetic tree in a human mtDNA study to investigate historical and evolutionary relationships.

### The hierBAPS-ML application

Studies of non-human species that utilized a hierBAPS-based phylogeny vary with respect to the description of the relationships between subclades and the genetic material being analyzed, for example, mtDNA [63] or chloroplast DNA and genomic markers [64–66]. These studies are typically supplemented by additional analyses, such as admixture and estimates of genetic diversity, or the addition of other biomarkers in the population, to draw inferences about their geographical dispersal [64–66].

A recent human mtDNA study used the non-hierarchical version of BAPS in its analysis to identify the origin and genetic affinities of Hill Tribes in Thailand with respect to other Asian populations [67], although a phylogenetic analysis was not undertaken in this study. After mapping the hierBAPS group within each specific population, the authors concluded that, although geographic neighbors were included within the same BAPS groups, it was not possible to draw any conclusions about the regional ancestry of the Hill Tribes. Similarly, mtDNA HVS-I sequences in African Brazilians have been analyzed using the same approach, although this analysis utilized hierBAPS to assess only basic population genetic structure, not the phylogenetic relationships among the sequences or the nested phylogenetic structure that hierBAPS provides [68].

While these studies assessed the genetic structure of the study populations, they were specifically limited in the ability to make evolutionary inferences about the lineages present in them. The incorporation of a rooted ML phylogeny facilitates making temporal inferences about the branching structure by mapping the progression of polymorphisms from an ancestral point-of-reference to the clusters found by the BAPS algorithm.

One of the greatest advantages of integrating hierBAPS algorithmic clustering with phylogenetic analysis is that it quickly disentangles relationships between large groups of similar sequences that would otherwise be difficult to interpret using haplogroup nomenclature alone. We have observed that the ability to distinguish between similar sequences was more specific when the mtDNA non-coding region was included, and less specific when it was removed. This outcome was expected, considering the high number of mutations that occur in the non-coding region of the human mitogenome [1]. Thus, with respect to mtDNA diversity, the greater the allelic information provided to the hierBAPS algorithm, the more detailed the resulting clustering.

### U5 Sequences in Northern Europe

The hierBAPS-ML analysis of haplogroup U5 was especially enhanced when combined with geographic information, age estimates, and U5 demographics. The results of this analysis confirmed a previous study of haplogroup U5 [25], which documented that subhaplogroup U5b1 expanded into Central and Southern Europe before it spread into Western Europe. Our results build upon this earlier study by focusing on the high frequency of U5 mtDNAs within the populations of the Scandinavian Peninsula and Finland, and exploring the geographic sources of the sequences that appear within the phylogeny of U5.

The hierBAPS-ML phylogeny showed that populations from Finland, Scandinavia, North Africa, and Central and Eastern Europe share several U5 subclades/hierBAPS groups. A previous study by Tambets and co-workers [32] found that the geographical source of the Saami-specific U5b1b1 subhaplogroup was difficult to discern. While haplogroup diversification in Southern and Western Europe indicated a west-to-east migration, the observation that the Saami-specific lineages were also present in Uralic-speaking populations of Eastern Europe [32] suggested that U5b1b may have arisen in and spread with these groups [32]. Our results supports the view that U5b1b divergence likely occurred via a scenario in which one subhaplogroup (U5b1b1) became prominent among African populations after hunter-gatherers crossed the Strait of Gibraltar [52]. The other subhaplogroup, U5b1b1a (subclade XVII) became prominent farther north in Scandinavia with the spread of U5b1b1, which eventually gave rise to the “Saami motif” [32, 37]. Furthermore, our phylogenetic tree showed that both lineages were distantly related to the younger subhaplogroups U5b1c and U5b1e1 in Central and Eastern Europe. This finding confirms that the migration of U5b1 mtDNAs likely occurred from west to east rather than the opposite direction.

Studies of the maternal lineages of Saami populations have focused on haplogroups U5b1b1 and V because they are found at the highest frequencies in these and other Scandinavian populations [32, 69]. While U5b1b1 comprises the vast majority of Saami U5 mtDNAs, other haplogroups in Saami populations may potentially have Southern and Central European sources. Lahermo and colleagues found a single U5b sequence, likely U5b3 based on its having the T16304C polymorphism, that was shared by Saami, Finns, and eastern-dwelling circumarctic populations [35]. Our analysis shows that this subhaplogroup is present among modern populations from Southern and Central Europe in addition to Scandinavia, indicating that it has a wide distribution. In fact, U5b3 is found at its highest frequency in Sardinia (3%), although it is the least frequent major U5 subhaplogroup in Europe (< 1% in most populations) [51].

Due to its proximity to Atlantic moisture, the Norwegian shelf was deglaciated between the local LGM and 14–10 thousand calibrated years before present (cal BP) [70], allowing migration from Southern Europe into Northern Europe to occur at that time. While Southern Europe became habitable for settlement during the Last Glacial Maximum, archeological evidence suggests there another co-existing refuge in the so-called “periglacial zone” was located in Ukraine and the West Siberian Plain [71]. Geological evidence supports this view, as ice retreat from the eastern portion of the Fennoscandinavian Ice Sheet led to the formation of large ice-dammed lakes separating the Baltic countries and Russia from Scandinavia [72], preventing early human migrations there. The Baltic Ice Lake persisted until approximately 11,620 ± 100 cal BP when dissipated, and before the time by which several U5 lineages had already started to expand [73].

Of these lineages, U5a2 constitutes a larger proportion of the U5 sequences in Eastern Europe, while there are also daughter branches of Group XXI containing Scandinavian, Finnish, and one Saami sequence. The earliest dispersals of U5a2 appear to have occurred in Central and Eastern Europe, with later dispersals into Scandinavia/Finland. We also note that some early U5a1 subhaplogroups (namely U5a1g and U5a1i) occur in the east. This second Ukrainian/Pontic refuge is a possible source of some U5 lineages having an eastern geographic origin.

The high frequency of U5, particularly U5b1 among the Saami, appears to be the result of genetic drift [31, 35, 74]. This interpretation is supported by a number of studies based on SNPs, and microsatellite markers which show a high level of linkage disequilibrium among the Saami [74–77] compared to surrounding Scandinavian populations. Most genetic studies further indicate that the Saami population formed as the result of several migration events into Fennoscandia through the coastal edges of land, after which the limited population size had minimally expanded over a long period of time [35, 78]. In this regard, Uralic speakers have been shown to have a distinct ancestral component of Siberian origin [79], with the Saami exhibiting a sizable proportion (13%) of East Eurasian ancestry [80].

It is not until the influx of haplogroups accompanying later dispersals during the Neolithic (approximately 11,000 – 6,500 kya) [81] that there is genetic evidence showing that the predominant U5 subhaplogroups had been diluted in Europe [82]. The Neolithic agriculturalists of central Europe carried mainly N1a, but also H, HV, J, K, T, V, and U3 haplogroups [83]. These Neolithic maternal lineages did not extend as successfully far north, where U5 comprises over 50% of maternal lineages among the Saami. Among Finns and Scandinavians, U5 continues to be the second-most frequent haplogroup after H [30, 31, 33].

Given its widespread distribution in Europe and especially northern Europe, there has been speculation about the possible adaptive features of haplogroup U5 mtDNAs. As an example, nonsynonymous substitutions identified in subclades U5a1 and U5a1a1b were found to arise at the time of maximal decrease in temperature, and suggested to reflect adaptive changes to the cytochrome b and ND5 gene in Europeans during the glaciation period [84]. That is, they were surmised to have produced more uncoupled mtDNAs that generate additional heat as a by-product of normal oxidative metabolism [84]. While these are intriguing results, more work is needed to demonstrate that these variants actually have this physiological effect.

From a clinical standpoint, haplogroup U5 has been linked to a number of complex diseases. For example, a case–control study of 406 patients and 183 healthy controls found a favorable statistical association between haplogroup U5 and the risk of cardiovascular infarction, but a higher risk of a low ventricular ejection fraction (< 40%) [85]. Another study found biological mechanisms that supported higher sperm motility among patients with U5 mtDNAs [86]. A third study found that the parent haplogroup U occurred at high frequency among patients with elevated risk for occipital brain infarct [87], with a related study suggesting that the association was due to a high frequency of haplogroup U5 [88]. Given that these findings are largely correlative in nature, verifying these associations and elucidating the mechanism by which this maternal lineage produces disease phenotypes will be needed to clarify the possible role of haplogroup U5 in human health and disease.

In conclusion, the combined hierBAPS-ML based phylogeny analysis provides insights into the phylogeographic partitioning of genetic diversity, providing a panoramic view of the range of subclades present. Further, it can quickly identify large subclades of related subhaplogroups for population studies that require analysis of a large number of individuals. Combined with archeological evidence, linguistic, and sociocultural knowledge, this methodology provides a visual consolidation of both ancestral and derived features of major mtDNA lineages that can enhance our understanding of human migration history.

## Supplementary Information

**Additional file 1:** **Supplementary material 1.** References to accompany Table S7

**Additional file 2:** **Figure S1.** Three analysis levels of hierBAPS groups superimposed onto a maximum likelihood phylogenic tree. **Figure S2.** Coding region only analysis of hierBAPS group identification using mtDNA. The hierBAPS groups have been superimposed on a phylogenetic tree, generated using maximum likelihood analysis to view the phylogenetic relationships of each sequence

**Additional file 3: Table S1. **Sensitivity analysis of hierBAPS groupings of human mtDNA U5 haplogroup using coding regions only. **Table S2.** Frequencies and proportions of mitochondrial DNA sequences used for hierBAPS-maximum likelihood evolutionary inferences. **Table S3.** The 24-level hierBAPS groups by geographic region. **Table S4.** Age estimates of the representative subclade(s) of the hierBAPS groups. **Table S5.** Age estimates and confidence intervals for Haplogroups in Figure 3. **Table S6.** Age estimates and confidence intervals for Haplogroups in Figure 4. **Table S7.** Percentage of U5 haplogroup within each population based on published literature in order of highest to lowest. **Table S8.** Mitochondrial DNA sequences used in this analysis.

## Data Availability

The mitogenome sequence data that are the focus of this study can be obtained from GenBank and the European Nucleotide Archive. Data from the European Nucleotide Archive are listed in project number PRJEB21940. The GenBank accession numbers for mitogenome sequences reported in this paper are listed in Table S8.

## Abbreviations

ML: Maximum likelihood
HierBAPS: Hierarchical Bayesian Analysis of Population Structure
Kya: Thousand years ago
RSRS: Reconstructed Sapiens Reference Sequence
Rcrs: Cambridge reference sequence
cal BP: Calibrated years before present
