# Correction: Evolution and dispersal of mitochondrial DNA haplogroup U5 in Northern Europe: insights from an unsupervised learning approach to phylogeography

**DOI:** 10.1186/s12864-022-08696-1

**Published:** 2022-06-21

**Authors:** Dana Kristjansson, Jon Bohlin, Truc Trung Nguyen, Astanand Jugessur, Theodore G. Schurr

**Affiliations:** 1 Center for Fertility and Health, Norwegian Institute of Public Health, Oslo, Norway; 2 Department of Global Public Health and Primary Care, Faculty of Medicine, University of Bergen, Bergen, Norway; 3 Department of Method Development and Analytics, Norwegian Institute of Public Health, Oslo, Norway; 4 IT Systems Bergen, Norwegian Institute of Public Health, Bergen, Norway; 5 Department of Anthropology, University of Pennsylvania, Philadelphia, PA USA

**Correction: BMC Genomics 23, 354 (2022)**

**https://doi.org/10.1186/s12864-022-08572-y**

Following publication of the original article [1], it was reported that a key in Fig. 5 contained an error. The colors designating ‘Group A: U5a1’ and ‘Group C: U5b1 and U5b3’ in the key ‘Haplogroup % based on 4 major hierBAPS groupings’ were reversed. The correct Fig. 5 is included in this Correction and the original article [1] has been updated.

**Fig. 5:**
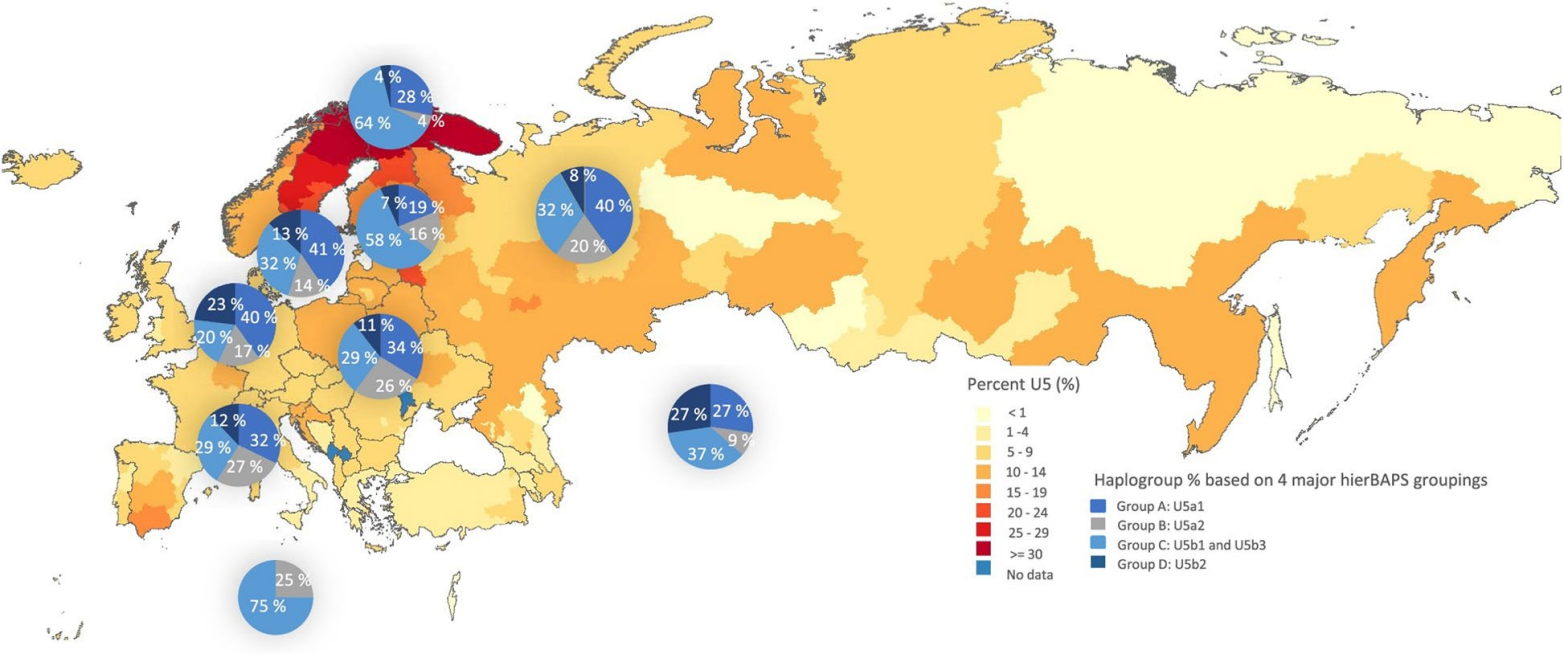
The frequency of haplogroup U5 mtDNAs in global populations based on the literature (see Table S3). The proportions of each subhaplogroup are listed, based on the four major hierBAPS groups from the FamilyTreeDNA’s U5 project. The sample sizes for each data set were as follows: Western Europe (*n*=537), Scandinavia (*n*=397), Sami (*n*=78), Finland (*n*=344), Southern Europe (*n*=124), Central Europe (*n*=166), and Eastern Europe (*n*=157). Countries within Asia (*n*=11) and Africa (*n*=4) were combined due to their small sample sizes, with the los frequency of U5 mtDNAs being supported by the literature (Table S3)
