# Supplementary material for: Evolution and dispersal of mitochondrial DNA haplogroup U5 in Northern Europe: insights from an unsupervised learning approach to phylogeography
Source: BMC Genomics. 2022 May 7;23:354. doi: 10.1186/s12864-022-08572-y (PMC9080151; doi:10.1186/s12864-022-08572-y)
Supplement: Supplementary file 1 — Additional file 1: Supplementary material 1. References to accompany Table S7 [file 12864_2022_8572_MOESM1_ESM.docx]

Supplementary Material 1. References to accompany Table S7.

Achilli A et al. 2007. Mitochondrial DNA variation of modern Tuscans supports the Near Eastern origin of Etruscans. Am. J. Hum. Genet. 80. doi: 10.1086/512822.

Al-Zahery N et al. 2003. Y-chromosome and mtDNA polymorphisms in Iraq, a crossroad of the early human dispersal and of post-Neolithic migrations. Mol. Phylogenet. Evol. 28. doi: 10.1016/S1055-7903(03)00039-3.

Alfonso-Sánchez MA et al. 2008. Mitochondrial DNA haplogroup diversity in basques: A reassessment based on HVI and HVII polymorphisms. Am. J. Hum. Biol. 20. doi: 10.1002/ajhb.20706.

Alfonso-Sánchez MA et al. 2006. Sequence polymorphisms of the mtDNA control region in a human isolate: The Georgians from Swanetia. J. Hum. Genet. 51. doi: 10.1007/s10038-006-0381-x.

Ahmic, A., Pojskic, N., Silajdzic, E., & Hadziselimovic, R. 2013. A preliminary study of the Paleolithic and Neolithic contribution the European mtdna flow in shaping the genetic structure of recent Bosnian population. *European Scientific Journal*, *9*(36).

Ahmić A, Hadžiselimović R, Silajdžić E, Mujkić I, Pojskić N. 2019. MtDNA variations in three main ethnic populations in Tuzla Canton of Bosnia and Herzegovina. Genet. Appl. 3. doi: 10.31383/ga.vol3iss1pp14-23.

Álvarez-Iglesias V et al. 2009. New population and phylogenetic features of the internal variation within mitochondrial DNA macro-haplogroup R0. PLoS One. 4. doi: 10.1371/journal.pone.0005112.

Baasner A, Schäfer C, Junge A, Madea B. 1998. Polymorphic sites in human mitochondrial DNA control region sequences: Population data and maternal inheritance. Forensic Sci. Int. 98. doi: 10.1016/S0379-0738(98)00163-7.

Babalini C et al. 2005. The population history of the Croatian linguistic minority of Molise (southern Italy): A maternal view. Eur. J. Hum. Genet. 13. doi: 10.1038/sj.ejhg.5201439.

Belledi M et al. 2000. Maternal and paternal lineages in Albania and the genetic structure of Indo-European populations. Eur. J. Hum. Genet. 8. doi: 10.1038/sj.ejhg.5200443.

Belyaeva O et al. 2003. Mitochondrial DNA Variations in Russian and Belorussian Populations. Hum. Biol. 75. doi: 10.1353/hub.2003.0069.

Barbarić L et al. 2020. Maternal perspective of Croatian genetic diversity. Forensic Sci. Int. Genet. 44. doi: 10.1016/j.fsigen.2019.102190.

Bermisheva MA et al. 2004. Phylogeographic analysis of mitochondrial DNA in the Nogays: A strong mixture of maternal lineages from Eastern and Western Eurasia. Mol. Biol. 38:516–523. doi: 10.1023/B:MBIL.0000037003.28999.45.

Bermisheva MA, Tambets K, Villeins R, Khusnutdinova EK. 2002. Diversity of mitochondrial DNA haplogroups in ethnic populations of the Volga-Ural region. Mol. Biol. 36. doi: 10.1023/A:1021677708482.

Bertranpetit J et al. 1995. Human mitochondrial DNA variation and the origin of Basques. Ann. Hum. Genet. 59. doi: 10.1111/j.1469-1809.1995.tb01606.x.

Bini C et al. 2003. Different informativeness of the three hypervariable mitochondrial DNA regions in the population of Bologna (Italy). Forensic Sci. Int. 135. doi: 10.1016/S0379-0738(03)00167-1.

Bogácsi-Szabó E et al. 2005. Mitochondrial DNA of ancient Cumanians: Culturally Asian steppe nomadic immigrants with substantially more western Eurasian mitochondrial DNA lineages. Hum. Biol. 77. doi: 10.1353/hub.2006.0007.

Bonné-Tamir B et al. 2003. Maternal and paternal lineages of the Samaritan isolate: Mutation rates and time to most recent common male ancestor. Ann. Hum. Genet. 67. doi: 10.1046/j.1469-1809.2003.00024.x.

Bosch E et al. 2006. Paternal and maternal lineages in the Balkans show a homogeneous landscape over linguistic barriers, except for the isolated Aromuns. Ann. Hum. Genet. 70. doi: 10.1111/j.1469-1809.2005.00251.x.

Brakez Z et al. 2001. Human mitochondrial DNA sequence variation in the Moroccan population of the Souss area. Ann. Hum. Biol. 28. doi: 10.1080/030144601300119106.

Brandstätter A, Klein R, Duftner N, Wiegand P, Parson W. 2006. Application of a quasi-median network analysis for the visualization of character conflicts to a population sample of mitochondrial DNA control region sequences from southern Germany (Ulm). Int. J. Legal Med. 120. doi: 10.1007/s00414-006-0114-x.

Brandstätter A, Niederstätter H, Pavlic M, Grubwieser P, Parson W. 2007. Generating population data for the EMPOP database-An overview of the mtDNA sequencing and data evaluation processes considering 273 Austrian control region sequences as example. Forensic Sci. Int. 166. doi: 10.1016/j.forsciint.2006.05.006.

Brehm A, Pereira L, Kivisild T, Amorim A. 2003. Mitochondrial portraits of the Madeira and Açores archipelagos witness different genetic pools of its settlers. Hum. Genet. 114. doi: 10.1007/s00439-003-1024-3.

Brisighelli F et al. 2012. Uniparental Markers of Contemporary Italian Population Reveals Details on Its Pre-Roman Heritage. PLoS One. 7. doi: 10.1371/journal.pone.0050794.

Calafell F, Underbill P, Tolun A, Angelicheva D, Kalaydjieva L. 1996. From Asia to Europe: Mitochondrial DNA sequence variability in Bulgarians and Turks. Ann. Hum. Genet. 60. doi: 10.1111/j.1469-1809.1996.tb01170.x.

Cali F et al. 2001. MtDNA control region and RFLP data for Sicily and France. Int. J. Legal Med. 114. doi: 10.1007/s004140000169.

Cardoso S et al. 2011. The maternal legacy of Basques in northern navarre: New insights into the mitochondrial DNA diversity of the Franco-Cantabrian area. Am. J. Phys. Anthropol. 145. doi: 10.1002/ajpa.21532.

Cardoso S et al. 2010. Variability of the entire mitochondrial DNA control region in a human isolate from the pas valley (Northern Spain). J. Forensic Sci. 55. doi: 10.1111/j.1556-4029.2010.01440.x.

Caruana J. 2012. Population Genetics of Western Mediterranean Islands - Malta: a case study. University of Manchester.

Černý V, Hájek M, Čmejla R, Brůžek J, Brdička R. 2004. mtDNA sequences of Chadic-speaking populations from northern Cameroon suggest their affinities with eastern Africa. Ann. Hum. Biol. 31. doi: 10.1080/03014460412331287182.

Cherni L et al. 2005. Female gene pools of Berber and Arab neighboring communities in Central Tunisia: Microstructure of mtDNA variation in North Africa. Hum. Biol. 77. doi: 10.1353/hub.2005.0028.

Cocoş R et al. 2017. Genetic affinities among the historical provinces of Romania and Central Europe as revealed by an mtDNA analysis. BMC Genet. 18. doi: 10.1186/s12863-017-0487-5.

Coia V et al. 2005. Brief Communication: mtDNA variation in North Cameroon: Lack of Asian lineages and implications for back migration from Asia to Sub-Saharan Africa. Am. J. Phys. Anthropol. 128. doi: 10.1002/ajpa.20138.

Coia V et al. 2012. Evidence of high genetic variation among linguistically diverse populations on a micro-geographic scale: A case study of the Italian Alps. J. Hum. Genet. 57. doi: 10.1038/jhg.2012.14.

Comas D et al. 2004. Admixture, migrations, and dispersals in Central Asia: Evidence from maternal DNA lineages. Eur. J. Hum. Genet. 12. doi: 10.1038/sj.ejhg.5201160.

Comas D et al. 1998. Trading genes along the silk road: mtDNA sequences and the origin of central Asian populations. Am. J. Hum. Genet. 63. doi: 10.1086/302133.

Comas D, Calafell F, Bendukidze N, Fañanás L, Bertranpetit J. 2000. Georgian and Kurd mtDNA sequence analysis shows a lack of correlation between languages and female genetic lineages. Am. J. Phys. Anthropol. 112. doi: 10.1002/(SICI)1096-8644(200005)112:1<5::AID-AJPA2>3.0.CO;2-Z.

Côrte-Real HBSM et al. 1996. Genetic diversity in the Iberian Peninsula determined from mitochondrial sequence analysis. Ann. Hum. Genet. 60:331–350. doi: 10.1111/j.1469-1809.1996.tb01196.x.

Crespillo M et al. 2000. Mitochondrial DNA sequences for 118 individuals from northeastern Spain. Int. J. Legal Med. 114. doi: 10.1007/s004140000158.

Cvjetan S et al. 2004. Frequencies of mtDNA haplogroups in Southeastern Europe - Croatians, Bosnians and Herzegovinians, Serbians, Macedonians and Macedonian Romani. Coll. Antropol. 28.

Davidovic S et al. 2015. Mitochondrial DNA perspective of serbian genetic diversity. Am. J. Phys. Anthropol. 156. doi: 10.1002/ajpa.22670.

Davidovic S et al. 2020. Complete mitogenome data for the Serbian population: the contribution to high-quality forensic databases. Int. J. Legal Med. 134. doi: 10.1007/s00414-020-02324-x.

Delghandi M, Utsi E, Krauss S. 1998. Saami mitochondrial DNA reveals deep maternal lineage clusters. Hum. Hered. 48. doi: 10.1159/000022789.

Derbeneva O. A., Starikovskaya EB, Volod’ko N V., Wallace DC, Sukernik RI. 2002. Mitochondrial DNA variation in Kets and Nganasans and the early peopling of Eastern Eurasia. Genetika. 38.

Derbeneva O. A., Starikovskaya EB, Volodko N V., Wallace DC, Sukernik RI. 2002. Mitochondrial DNA Variation in the Kets and Nganasans and Its Implications for the Initial Peopling of Northern Eurasia. Russ. J. Genet. 38. doi: 10.1023/A:1021111530654.

Derbeneva Olga A., Starikovskaya EB, Wallace DC, Sukernik RI. 2002. Traces of early Eurasians in the Mansi of northwest Siberia revealed by mitochondrial DNA analysis. Am. J. Hum. Genet. 70. doi: 10.1086/339524.

Destro-Bisol G et al. 2004. The analysis of variation of mtDNA hypervariable region 1 suggests that Eastern and Western Pygmies diverged before the Bantu expansion. Am. Nat. 163. doi: 10.1086/381405.

Di Benedetto G et al. 2001. DNA diversity and population admixture in Anatolia. Am. J. Phys. Anthropol. 115. doi: 10.1002/ajpa.1064.

Dimo-Simonin N, Grange F, Taroni F, Brandt-Casadevall C, Mangin P. 2000. Forensic evaluation of mtDNA in a population from south west Switzerland. Int. J. Legal Med. 113. doi: 10.1007/PL00007715.

Di Rienzo A, Wilson AC. 1991. Branching pattern in the evolutionary tree for human mitochondrial DNA. Proc. Natl. Acad. Sci. U. S. A. 88. doi: 10.1073/pnas.88.5.1597.

Dubut V et al. 2004. mtDNA polymorphisms in five French groups: Importance of regional sampling. Eur. J. Hum. Genet. 12. doi: 10.1038/sj.ejhg.5201145.

Dupuy BM, Olaisen B. 1996. mtDNA sequences in the Norwegian Saami and main populations. In: Advances in Forensic Haemogenetics. Carracedo A., Brinkmann B., BW, editor. Springer: Berlin, Heidelberg pp. 23–25. doi: 10.1007/978-3-642-80029-0_6.

Eduardoff M et al. 2013. Mass spectrometric base composition profiling: Implications for forensic mtDNA databasing. Forensic Sci. Int. Genet. 7. doi: 10.1016/j.fsigen.2013.05.007.

Fadhlaoui-Zid K et al. 2004. Mitochondrial DNA heterogeneity in Tunisian Berbers. Ann. Hum. Genet. 68. doi: 10.1046/j.1529-8817.2004.00096.x.

Falchi A et al. 2006. Genetic history of some western Mediterranean human isolates through mtDNA HVR1 polymorphisms. J. Hum. Genet. 51. doi: 10.1007/s10038-005-0324-y.

FamilyTreeDNA. 2021a. Family Tree DNA - The Saami Project. FamilyTreeDNA. https://www.familytreedna.com/groups/saami/about (Accessed May 31, 2021).

FamilyTreeDNA. 2021b. FamilyTreeDNA - The U5 Project. FamilyTreeDNA.

Fedorova SA, Bermisheva MA, Villems R, Maksimova NR, Khusnutdinova EK. 2003. Analysis of Mitochondrial DNA Lineages in Yakuts. Mol. Biol. 37. doi: 10.1023/A:1025135326954.

Francalacci P, Bertranpetit J, Calafell F, Underhill PA. 1996. Sequence diversity of the control region of mitochondrial DNA in Tuscany and its implications for the peopling of Europe. Am. J. Phys. Anthropol. 100. doi: 10.1002/(SICI)1096-8644(199608)100:4<443::AID-AJPA1>3.0.CO;2-S.

Fraumene C et al. 2006. High resolution analysis and phylogenetic network construction using complete mtDNA sequences in Sardinian genetic isolates. Mol. Biol. Evol. 23. doi: 10.1093/molbev/msl084.

García O et al. 2011. Using mitochondrial DNA to test the hypothesis of a European post-glacial human recolonization from the Franco-Cantabrian refuge. Heredity (Edinb). 106. doi: 10.1038/hdy.2010.47.

Gonzalez AM et al. 2003. Mitochondrial DNA affinities at the atlantic fringe of Europe. Am. J. Phys. Anthropol. 120. doi: 10.1002/ajpa.10168.

Goodacre S et al. 2005. Genetic evidence for a family-based Scandinavian settlement of Shetland and Orkney during the Viking periods. Heredity (Edinb). 95. doi: 10.1038/sj.hdy.6800661.

Gómez-Carballa A, Pardo-Seco J, Amigo J, Martinón-Torres F, Salas A. 2015. Mitogenomes from The 1000 Genome Project reveal new Near Eastern features in present-day Tuscans. PLoS One. 10. doi: 10.1371/journal.pone.0119242.

Graven L et al. 1995. Evolutionary correlation between control region sequence and restriction polymorphisms in the mitochondrial genome of a large senegalese mandenka sample. Mol. Biol. Evol. 12. doi: 10.1093/oxfordjournals.molbev.a040206.

Grosheva AN, Shneider Y V., Zhukova O V., Morozova IY, Rychkov SY. 2014. Features of the Udmurt mitochondrial gene pool in relation to tribal structure. Russ. J. Genet. 50. doi: 10.1134/S1022795414090063.

Grzybowski T et al. 2007. Complex interactions of the Eastern and Western Slavic populations with other European groups as revealed by mitochondrial DNA analysis. Forensic Sci. Int. Genet. 1. doi: 10.1016/j.fsigen.2007.01.010.

Gubina MA, Damba LD, Babenko VN, Romaschenko AG, Voevoda MI. 2013. Haplotype diversity in mtDNA and Y-chromosome in populations of Altai-Sayan region. Russ. J. Genet. 49. doi: 10.1134/S1022795412120034.

Hedman M et al. 2007. Finnish mitochondrial DNA HVS-I and HVS-II population data. Forensic Sci. Int. 172. doi: 10.1016/j.forsciint.2006.09.012.

Helgason A et al. 2001. mtDNA and the Islands of the North Atlantic: Estimating the proportions of Norse and Gaelic ancestry. Am. J. Hum. Genet. 68:723–737. doi: 10.1086/318785.

Hernández CL et al. 2014. Human maternal heritage in Andalusia (Spain): Its composition reveals high internal complexity and distinctive influences of mtDNA haplogroups U6 and L in the western and eastern side of region. BMC Genet. 15. doi: 10.1186/1471-2156-15-11.

Hervella M et al. 2014. The Carpathian range represents a weak genetic barrier in South-East Europe. BMC Genet. 15. doi: 10.1186/1471-2156-15-56.

Hofmann S et al. 1997. Population genetics and disease susceptibility: Characterization of central European haplogroups by mtDNA gene mutations, correlation with D loop variants and association with disease. Hum. Mol. Genet. 6. doi: 10.1093/hmg/6.11.1835.

Irwin J et al. 2007. Hungarian mtDNA population databases from Budapest and the Baranya county Roma. Int. J. Legal Med. 121. doi: 10.1007/s00414-006-0128-4.

Irwin J et al. 2008. Mitochondrial control region sequences from northern Greece and Greek Cypriots. Int. J. Legal Med. 122. doi: 10.1007/s00414-007-0173-7.

Jackson BA et al. 2005. Mitochondrial DNA genetic diversity among four ethnic groups in Sierra Leone. Am. J. Phys. Anthropol. 128. doi: 10.1002/ajpa.20040.

Jankova-Ajanovska R et al. 2014. Mitochondrial DNA control region analysis of three ethnic groups in the Republic of Macedonia. Forensic Sci. Int. Genet. 13. doi: 10.1016/j.fsigen.2014.06.013.

Jarczak J et al. 2019. Mitochondrial DNA variability of the Polish population. Eur. J. Hum. Genet. 27. doi: 10.1038/s41431-019-0381-x.

Kasperavičiute D, Kučinskas V, Stoneking M. 2004. Y chromosome and mitochondrial DNA variation in Lithuanians. Ann. Hum. Genet. 68. doi: 10.1046/j.1529-8817.2003.00119.x.

Kittles RA et al. 1999. Autosomal, mitochondrial, and Y chromosome DNA variation in Finland: Evidence for a male-specific bottleneck. Am. J. Phys. Anthropol. 108:381–399. doi: 10.1002/(SICI)1096-8644(199904)108:4<381::AID-AJPA1>3.0.CO;2-5.

Karachanak S et al. 2012. Bulgarians vs the other European populations: A mitochondrial DNA perspective. Int. J. Legal Med. 126. doi: 10.1007/s00414-011-0589-y.

Kivisild T et al. 1999. Deep common ancestry of indian and western-Eurasian mitochondrial DNA lineages. Curr. Biol. 9. doi: 10.1016/S0960-9822(00)80057-3.

Kivisild T et al. 2004. Ethiopian mitochondrial DNA heritage: Tracking gene flow across and around the gate of tears. Am. J. Hum. Genet. 75. doi: 10.1086/425161.

Kouvatsi A, Karaiskou N, Apostolidis A, Kirmizidis G. 2001. Mitochondrial DNA sequence variation in Greeks. Hum. Biol. 73. doi: 10.1353/hub.2001.0085.

Kovacevic L et al. 2014. Standing at the gateway to Europe - The genetic structure of Western Balkan populations based on autosomal and haploid markers. PLoS One. 9. doi: 10.1371/journal.pone.0105090.

Krings M et al. 1999. mtDNA analysis of Nile River Valley populations: A genetic corridor or a barrier to migration? Am. J. Hum. Genet. 64. doi: 10.1086/302314.

Kushniarevich A et al. 2013. Uniparental Genetic Heritage of Belarusians: Encounter of Rare Middle Eastern Matrilineages with a Central European Mitochondrial DNA Pool. PLoS One. 8. doi: 10.1371/journal.pone.0066499.

Lahermo P et al. 2000. MtDNA polymorphism in the Hungarians: Comparison to three other Finno-Ugric-speaking populations. Hereditas. 132. doi: 10.1111/j.1601-5223.2000.00035.x.

Lahermo P et al. 1996. The genetic relationship between the Finns and the Finnish Saami (Lapps): Analysis of nuclear DNA and mtDNA. Am. J. Hum. Genet. 58:1309–1322. /pmc/articles/PMC1915079/?report=abstract (Accessed February 11, 2021).

Lappalainen T et al. 2008. Migration waves to the baltic sea region. Ann. Hum. Genet. 72. doi: 10.1111/j.1469-1809.2007.00429.x.

Larruga JM, Díez F, Pinto FM, Flores C, González AM. 2001. Mitochondrial DNA characterisation of European isolates: The Maragatos from Spain. Eur. J. Hum. Genet. 9. doi: 10.1038/sj.ejhg.5200693.

Lehocký I, Baldovič M, Kádaši Ľ, Metspalu E. 2008. A database of mitochondrial DNA hypervariable regions I and II sequences of individuals from Slovakia. Forensic Sci. Int. Genet. 2. doi: 10.1016/j.fsigen.2007.12.008.

Lembring M, Van Oven M, Montelius M, Allen M. 2013. Mitochondrial DNA analysis of Swedish population samples. Int. J. Legal Med. 127:1097–1099. doi: 10.1007/s00414-013-0908-6.

Lutz S, Weisser HJ, Heizmann J, Pollak S. 1998. Location and frequency of polymorphic positions in the mtDNA control region of individuals from Germany. Int. J. Legal Med. 111. doi: 10.1007/s004140050117.

Maca-Meyer N et al. 2003. Y chromosome and mitochondrial DNA characterization of Pasiegos, a human isolate from Cantabria (Spain). Ann. Hum. Genet. 67. doi: 10.1046/j.1469-1809.2003.00045.x.

Mairal Q et al. 2013. Linguistic isolates in Portugal: Insights from the mitochondrial DNA pattern. Forensic Sci. Int. Genet. 7. doi: 10.1016/j.fsigen.2013.08.009.

Malyarchuk BA, Derenko M V. 2001. Mitochondrial DNA variability in Russians and Ukrainians: Implication to the origin of the Eastern Slavs. Ann. Hum. Genet. doi: 10.1046/j.1469-1809.2001.6510063.x.

Malyarchuk BA et al. 2002. Mitochondrial DNA variability in Poles and Russians. Ann. Hum. Genet. 66. doi: 10.1017/S0003480002001161.

Malyarchuk B et al. 2004. Differentiation of mitochondrial DNA and Y chromosomes in Russian populations. Hum. Biol. 76:877–900. doi: 10.1353/hub.2005.0021.

Malyarchuk BA et al. 2003. Mitochondrial DNA variability in Bosnians and Slovenians. Ann. Hum. Genet. 67. doi: 10.1046/j.1469-1809.2003.00042.x.

Malyarchuk BA, Vanecek T, Perkova MA, Derenko M V., Sip M. 2006. Mitochondrial DNA variability in the Czech population, with application to the ethnic history of Slavs. Hum. Biol. 78. doi: 10.1353/hub.2007.0014.

Malyarchuk BA et al. 2008. Reconstructing the phylogeny of African mitochondrial DNA lineages in Slavs. Eur. J. Hum. Genet. 16. doi: 10.1038/ejhg.2008.70.

Mateu E et al. 1997. A tale of two islands: Population history and mitochondrial DNA sequence variation of Bioko and Sao Tome, Gulf of Guinea. Ann. Hum. Genet. 61. doi: 10.1017/S0003480097006544.

Meinilä M, Finnilä S, Majamaa K. 2001. Evidence for mtDNA admixture between the Finns and the Saami. Hum. Hered. 52:160–170. doi: 10.1159/000053372.

Messina F, Scorrano G, Labarga CM, Rolfo MF, Rickards O. 2010. Mitochondrial DNA variation in an isolated area of Central Italy. Ann. Hum. Biol. 37. doi: 10.3109/03014461003720304.

Metspalu M et al. 2004. Most of the extant mtDNA boundaries in South and Southwest Asia were likely shaped during the initial settlement of Eurasia by anatomically modern humans. BMC Genet. 5. doi: 10.1186/1471-2156-5-26.

Mergen H, Öner R, Öner C. 2004. Mitochondrial DNA sequence variation in the Anatolian peninsula (Turkey). J. Genet. 83. doi: 10.1007/BF02715828.

Mielnik-Sikorska M et al. 2013. The History of Slavs Inferred from Complete Mitochondrial Genome Sequences Pereira, LMSM, editor. PLoS One. 8:e54360. doi: 10.1371/journal.pone.0054360.

Mikkelsen M, Sørensen E, Rasmussen EM, Morling N. 2010. Mitochondrial DNA HV1 and HV2 variation in Danes. Forensic Sci. Int. Genet. 4. doi: 10.1016/j.fsigen.2009.07.007.

Modi A et al. 2020. The mitogenome portrait of Umbria in Central Italy as depicted by contemporary inhabitants and pre-Roman remains. Sci. Rep. 10. doi: 10.1038/s41598-020-67445-0.

Mogentale-Profizi N et al. 2001. Mitochondrial DNA sequence diversity in two groups of Italian Veneto speakers from Veneto. Ann. Hum. Genet. 65. doi: 10.1017/S0003480001008545.

Morelli L et al. 2000. Frequency distribution of mitochondrial DNA haplogroups in Corsica and Sardinia. Hum. Biol. 72.

Morozova I et al. 2012. Russian ethnic history inferred from mitochondrial DNA diversity. Am. J. Phys. Anthropol. 147. doi: 10.1002/ajpa.21649.

Nasidze I et al. 2005. Genetic evidence for the Mongolian ancestry of Kalmyks. Am. J. Phys. Anthropol. 128. doi: 10.1002/ajpa.20159.

Nasidze I, Quinque D, Rahmani M, Alemohamad SA, Stoneking M. 2006. Concomitant Replacement of Language and mtDNA in South Caspian Populations of Iran. Curr. Biol. 16. doi: 10.1016/j.cub.2006.02.021.

Nasidze I, Stoneking M. 2001. Mitochondrial DNA variation and language replacements in the Caucasus. Proc. R. Soc. B Biol. Sci. 268. doi: 10.1098/rspb.2001.1610.

Opdal SHS et al. Increased number of substitutions in the D-loop of mitochondrial DNA in the sudden infant death syndrome. 87:1039–1044. http://doi.wiley.com/10.1111/j.1651-2227.1998.tb01410.x (Accessed January 11, 2021).

Orekhov V et al. 1999. Mitochondrial DNA sequence diversity in Russians. FEBS Lett. 445. doi: 10.1016/S0014-5793(99)00115-5.

Ottoni C et al. 2009. Human mitochondrial DNA variation in Southern Italy. Ann. Hum. Biol. 36. doi: 10.3109/03014460903198509.

Parson W, Parsons TJ, Scheithauer R, Holland MM. 1998. Population data for 101 Austrian Caucasian mitochondrial DNA d-loop sequences: Application of mtDNA sequence analysis to a forensic case. Int. J. Legal Med. 111. doi: 10.1007/s004140050132.

Passarino G et al. 2002. Different genetic components in the Norwegian population revealed by the analysis of mtDNA and Y chromosome polymorphisms. Eur. J. Hum. Genet. 10:521–529. doi: 10.1038/sj.ejhg.5200834.

Pardiñas AF, Roca A, Garcia-Vazquez E, Lopez B. 2012. Mitochondrial diversity patterns and the Magdalenian resettlement of Europe: New insights from the edge of the Franco-Cantabrian refuge. J. Hum. Genet. 57. doi: 10.1038/jhg.2012.100.

Pereira V, Gomes V, Amorim A, Gusmão L, Prata MJ. 2010. Genetic characterization of uniparental lineages in populations from Southwest Iberia with past malaria endemicity. Am. J. Hum. Biol. 22. doi: 10.1002/ajhb.21049.

Pereira L et al. 2005. High-resolution mtDNA evidence for the late-glacial resettlement of Europe from an Iberian refugium. Genome Res. 15. doi: 10.1101/gr.3182305.

Piercy R, Sullivan KM, Benson N, Gill P. 1993. The application of mitochondrial DNA typing to the study of white Caucasian genetic identification. Int. J. Legal Med. 106. doi: 10.1007/BF01225046.

Picornell A, Gómez-Barbeito L, Tomàs C, Castro JA, Ramon MM. 2005. Mitochondrial DNA HVRI variation in Balearic populations. Am. J. Phys. Anthropol. 128. doi: 10.1002/ajpa.10423.

Plaza S et al. 2003. Joining the pillars of hercules: mtDNA sequences show multidirectional gene flow in the Western Mediterranean. Ann. Hum. Genet. 67. doi: 10.1046/j.1469-1809.2003.00039.x.

Pliss L et al. 2006. Mitochondrial DNA portrait of Latvians: Towards the understanding of the genetic structure of Baltic-speaking populations. Ann. Hum. Genet. 70. doi: 10.1111/j.1469-1809.2005.00238.x.

Poetsch M, Wittig H, Krause D, Lignitz E. 2003. Mitochondrial diversity of a northeast German population sample. Forensic Sci. Int. 137. doi: 10.1016/j.forsciint.2003.06.001.

Prieto L et al. 2011. The GHEP-EMPOP collaboration on mtDNA population data - A new resource for forensic casework. Forensic Sci. Int. Genet. 5. doi: 10.1016/j.fsigen.2010.10.013.

Pshenichnov A et al. 2013. Genetic affinities of Ukrainians from the maternal perspective. Am. J. Phys. Anthropol. 152. doi: 10.1002/ajpa.22371.

Pult I et al. 1994. Mitochondrial DNA sequences from Switzerland reveal striking homogeneity of European populations. Biol. Chem. Hoppe. Seyler. 375. doi: 10.1515/bchm3.1994.375.12.837.

Quintana-Murci L et al. 2004. Where West Meets East: The Complex mtDNA Landscape of the Southwest and Central Asian Corridor. Am. J. Hum. Genet. 74. doi: 10.1086/383236.

Rando JC et al. 1998. Mitochondrial DNA analysis of Northwest African populations reveals genetic exchanges with European, Near-Eastern, and sub-Saharan populations. Ann. Hum. Genet. 62. doi: 10.1017/S0003480099007241.

Richard C et al. 2007. An mtDNA perspective of French genetic variation. Ann. Hum. Biol. 34:68–79. doi: 10.1080/03014460601076098.

Richards M et al. 1996. Paleolithic and neolithic lineages in the European mitochondrial gene pool. Am. J. Hum. Genet. 59:185. doi: 10.1086/516858.

Richards M et al. 2000. Tracing european founder lineages in the near eastern mtDNA pool. Am. J. Hum. Genet. 67:1251–1276. doi: 10.1016/S0002-9297(07)62954-1.

Rousselet F, Mangin P. 1998. Mitochondrial DNA polymorphisms: A study of 50 French Caucasian individuals and application to forensic casework. Int. J. Legal Med. 111. doi: 10.1007/s004140050174.

Sajantila A et al. 1995. Genes and languages in Europe: An analysis of mitochondrial lineages. Genome Res. 5:42–52. doi: 10.1101/gr.5.1.42.

Sajantila A et al. 1996. Paternal and maternal DNA lineages reveal a bottleneck in the founding of the Finnish population. Proc. Natl. Acad. Sci. U. S. A. 93. doi: 10.1073/pnas.93.21.12035.

Santos C et al. 2003. Genetic structure and origin of peopling in the Azores Islands (Portugal): The view from mtDNA. Ann. Hum. Genet. 67. doi: 10.1046/j.1469-1809.2003.00031.x.

Schönberg A, Theunert C, Li M, Stoneking M, Nasidze I. 2011. High-throughput sequencing of complete human mtDNA genomes from the Caucasus and West Asia: High diversity and demographic inferences. Eur. J. Hum. Genet. 19. doi: 10.1038/ejhg.2011.62.

Seehausen O et al. 2003. Nuclear markers reveal unexpected genetic variation and a Congolese-Nilotic origin of the Lake Victoria cichlid species flock. Proc. R. Soc. B Biol. Sci. 270. doi: 10.1098/rspb.2002.2153.

Šarac J et al. 2014. Maternal genetic heritage of southeastern europe reveals a new croatian isolate and a novel, local sub-branching in the X2 haplogroup. Ann. Hum. Genet. 78. doi: 10.1111/ahg.12056.

Simoni L, Calafell F, Pettener D, Bertranpetit J, Barbujani G. 2000. Geographic patterns of mtDNA diversity in Europe. Am. J. Hum. Genet. 66:262–278. doi: 10.1086/302706.

Stenico M et al. 1996. High mitochondrial sequence diversity in linguistic isolates of the Alps. Am. J. Hum. Genet. 59.

Stevanovitch A et al. 2004. Mitochondrial DNA sequence diversity in a sedentary population from Egypt. Ann. Hum. Genet. 68. doi: 10.1046/j.1529-8817.2003.00057.x.

Stoljarova M, King JL, Takahashi M, Aaspõllu A, Budowle B. 2016. Whole mitochondrial genome genetic diversity in an Estonian population sample. Int. J. Legal Med. 130. doi: 10.1007/s00414-015-1249-4.

Sykes B. 2006. Saxons, Vikings, and Celts: The Genetic Roots of Britain and Ireland. In: W. W. Norton and company: New York pp. 147–164.

Tagliabracci A, Turchi C, Buscemi L, Sassaroli C. 2001. Polymorphism of the mitochondrial DNA control region in Italians. Int. J. Legal Med. 114. doi: 10.1007/s004140000168.

Tambets K et al. 2004. The western and eastern roots of the Saami - the story of genetic ‘outliers’ told by mitochondrial DNA and Y chromosomes. Am. J. Hum. Genet. 74:661–682. doi: 10.1086/383203.

Tetzlaff S, Brandstätter A, Wegener R, Parson W, Weirich V. 2007. Mitochondrial DNA population data of HVS-I and HVS-II sequences from a northeast German sample. Forensic Sci. Int. 172. doi: 10.1016/j.forsciint.2006.12.016.

Tillmar AO, Coble MD, Wallerström T, Holmlund G. 2010. Homogeneity in mitochondrial DNA control region sequences in Swedish subpopulations. Int. J. Legal Med. 124. doi: 10.1007/s00414-009-0354-7.

Tolk H V. et al. 2000. MtDNA haplogroups in the populations of Croatian Adriatic islands. Coll. Antropol. 24.

Tömöry G et al. 2007. Comparison of maternal lineage and biogeographic analyses of ancient and modern Hungarian populations. Am. J. Phys. Anthropol. 134. doi: 10.1002/ajpa.20677.

Tonks S, Winney B, Evseeva I. 2006. Comparison of Sex-Linked and Autosomal Markers in Orkney and Other North European Populations. Genbank data. www.ncbi.nlm.nih.gov (Accessed May 31, 2021).

Turchi C et al. 2008. Italian mitochondrial DNA database: Results of a collaborative exercise and proficiency testing. Int. J. Legal Med. 122. doi: 10.1007/s00414-007-0207-1.

Turchi C et al. 2016. The mitochondrial DNA makeup of Romanians: A forensic mtDNA control region database and phylogenetic characterization. Forensic Sci. Int. Genet. 24. doi: 10.1016/j.fsigen.2016.06.013.

Varesi L et al. 2000. Mitochondrial control-region sequence variation in the Corsican population, France. Am. J. Hum. Biol. 12. doi: 10.1002/(sici)1520-6300(200005/06)12:3<339::aid-ajhb4>3.0.co;2-u.

Vidrová V et al. 2008. Mitochondrial DNA haplogroups in the Czech population compared to other European countries. Hum. Biol. 80. doi: 10.3378/1534-6617-80.6.669.

Vigilant L, Stoneking M, Harpending H, Hawkes K, Wilson AC. 1991. African populations and the evolution of human mitochondrial DNA. Science (80-. ). 253. doi: 10.1126/science.1840702.

Vona G et al. 2001. Mitochondrial DNA sequence analysis in Sicily. Am. J. Hum. Biol. 13. doi: 10.1002/ajhb.1096.

Watson E et al. 1996. mtDNA sequence diversity in Africa. Am. J. Hum. Genet. 59.

Zgonjanin D et al. 2010. Sequence polymorphism of the mitochondrial DNA control region in the population of Vojvodina Province, Serbia. Leg. Med. 12. doi: 10.1016/j.legalmed.2009.10.007.

Zimmermann B et al. 2007. Mitochondrial DNA control region population data from Macedonia. Forensic Sci. Int. Genet. 1. doi: 10.1016/j.fsigen.2007.03.002.

Zupan A, Hauptman N, Glavač D. 2016. The maternal perspective for five Slovenian regions: The importance of regional sampling. Ann. Hum. Biol. 43. doi: 10.3109/03014460.2015.1006678.
