# Supplementary material for: Evolution and dispersal of mitochondrial DNA haplogroup U5 in Northern Europe: insights from an unsupervised learning approach to phylogeography
Source: BMC Genomics. 2022 May 7;23:354. doi: 10.1186/s12864-022-08572-y (PMC9080151; doi:10.1186/s12864-022-08572-y)
Supplement: Supplementary file 2 — Additional file 2: Figure S1. Three analysis levels of hierBAPS groups superimposed onto a maximum likelihood phylogenic tree. Figure S2. Coding region only analysis of hierBAPS group identification using mtDNA. The hierBAPS groups have been superimposed on a phylogenetic tree, generated using maximum likelihood analysis to view the phylogenetic relationships of each sequence [file 12864_2022_8572_MOESM2_ESM.pdf]

Figure S1

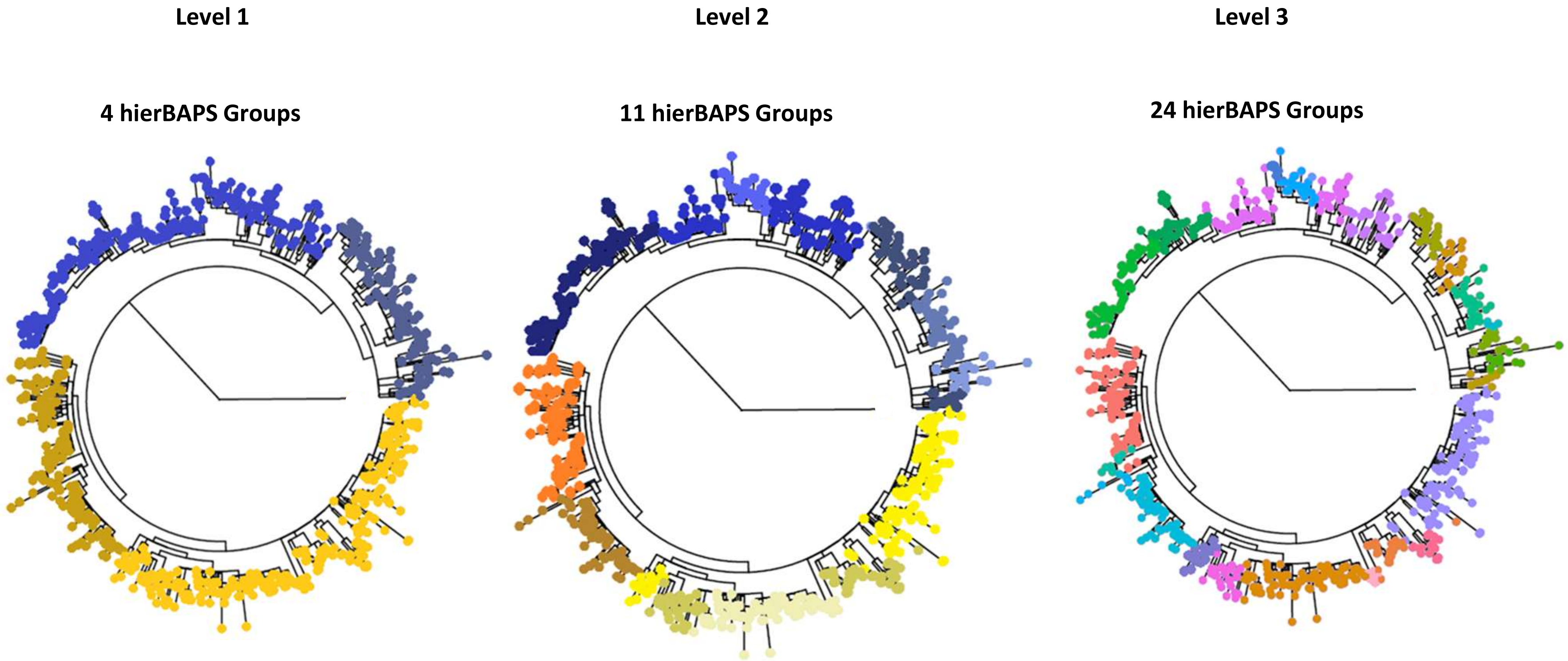

Figure S1. Three analysis levels of hierBAPS groups superimposed onto a maximum likelihood phylogenic tree.

Figure S2

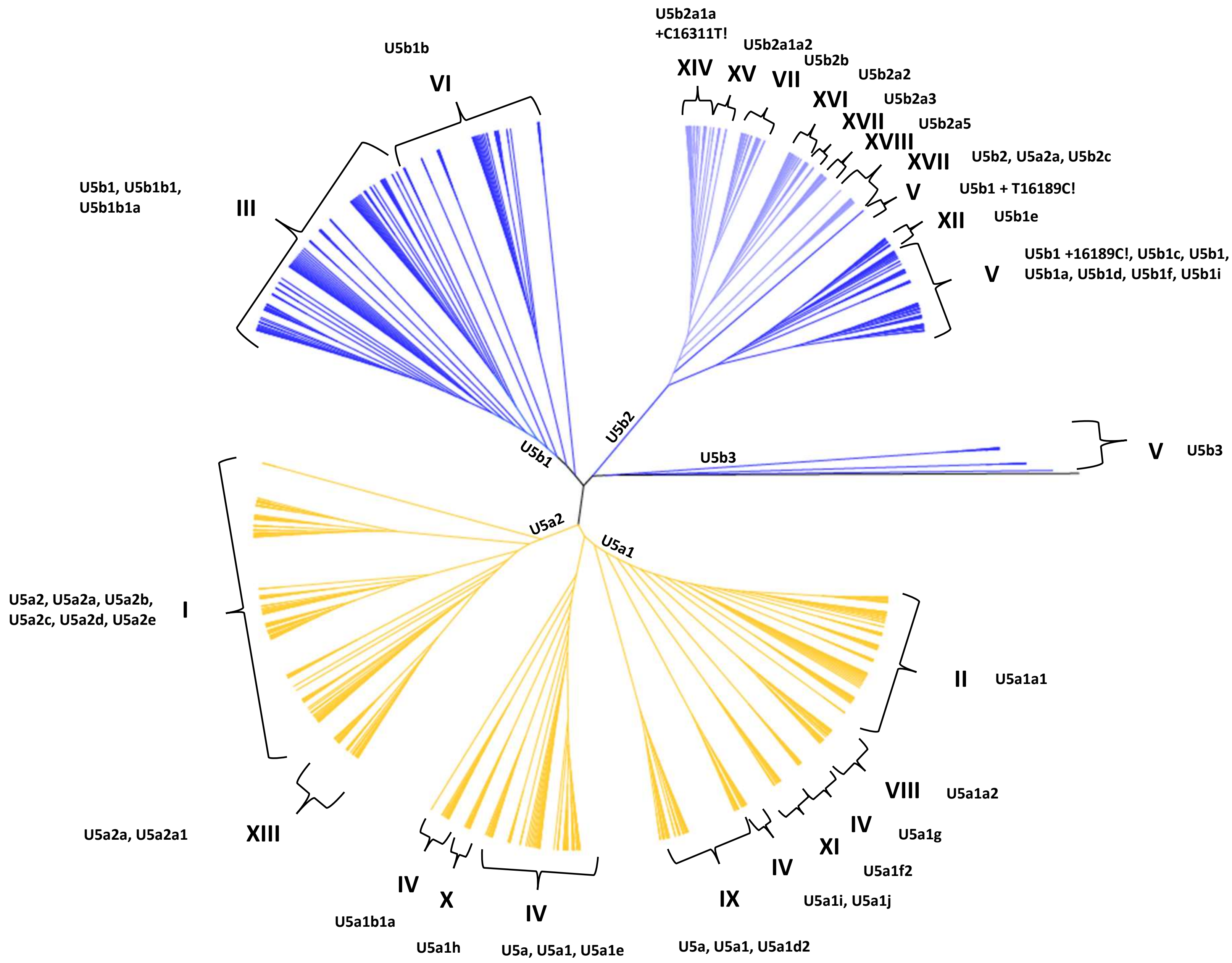

Figure S2. Coding region only analysis of hierBAPS group identification using mtDNA. The hierBAPS groups have been superimposed on a phylogenetic tree, generated using maximum likelihood analysis to view the phylogenetic relationships of each sequence.
